# Supplementary material for: How geographic access to care shapes disease burden: The current impact of post-exposure prophylaxis and potential for expanded access to prevent human rabies deaths in Madagascar
Source: PLoS Negl Trop Dis. 2021 Apr 26;15(4):e0008821. doi: 10.1371/journal.pntd.0008821 (PMC8102000; doi:10.1371/journal.pntd.0008821)
Supplement: S1 Text — Fig A. Raster inputs to estimate travel times to the closest clinic provisioning PEP for Madagascar. (A) Friction surface of travel speeds (in minutes per meter) at an ~ 1x1 km scale, with location of current clinics provisioning PEP (N = 31) shown with black x’s (original source: Malaria Atlas Project friction surface, (https://malariaatlas.org/research-project/accessibility-to-cities/, CC-BY 3.0). (B) Population estimates resampled to the same friction surface (original source: WorldPop, https://www.worldpop.org/geodata/summary?id=70, CC-BY 4.0 license). Fig B. Raw patient reported and driving travel time data. (A) Distribution of travel times estimated at the grid cell level and reported by patients for each commune where patient data were available from the Moramanga PEP clinic (B) Reported driving times between locations, with the color corresponding to the total driving time and the size of the line showing the direction of travel (narrow to wide ~ origin to destination). Paths are Bezier curves from origin to destination, and do not show actual paths driven. Administrative boundaries from OCHA via HDX (https://data.humdata.org/dataset/madagascar-administrative-level-0-4-boundaries, CC-BY-IGO). Fig C. Reported modes of transport used compared to reported travel times for patients reporting to the Moramanga PEP clinic. Fig D. Observed estimates of travel times (commune means of patient reported travel times and driving times between point locations) vs. estimates from friction surface. Predicted by (A) Distance (km) (Euclidean distance between origin and destination for driving times and distance from the commune centroid to the Moramanga PEP clinic for commune means) (B) Travel time estimates and (C) Travel time estimates weighted by population (for commune means only). Administrative boundaries are from OCHA (https://data.humdata.org/dataset/madagascar-administrative-level-0-4-boundaries, CC-BY-IGO). Table A. R2 values from linear models with estimated access [file pntd.0008821.s001.pdf]

S1 Text. Estimating travel times to the closest clinic provisioning PEP

How geographic access to care shapes disease burden: the current impact of post-exposure prophylaxis and potential for expanded access to prevent human rabies deaths in Madagascar

Rajeev et al. 2021

Table of Contents

Fig A. Raster inputs to estimate travel times to the closest clinic provisioning PEP for Madagascar .. 2
Fig B. Raw patient reported and driving travel time data. ... 3
Fig C. Reported modes of transport used compared to reported travel times for patients reporting to the Moramanga PEP clinic ... 4
Fig D. Observed estimates of travel times (commune means of patient reported travel times and driving times between point locations) vs. estimates from friction surface. ... 5
Table A. R^2 values from linear models with estimated access metrics predicting either driving times or commune means of patient reported travel times. ... 6
Fig E. Estimates of mean travel times weighted by population at the (A) District (B) Commune scale. Administrative boundaries from OCHA via HDX (https://data.humdata.org/dataset/madagascar-administrative-level-0-4-boundaries, CC-BY-IGO). ... 7

Correspondence: Rajeev et al. 2021 <mrajeev@princeton.edu>

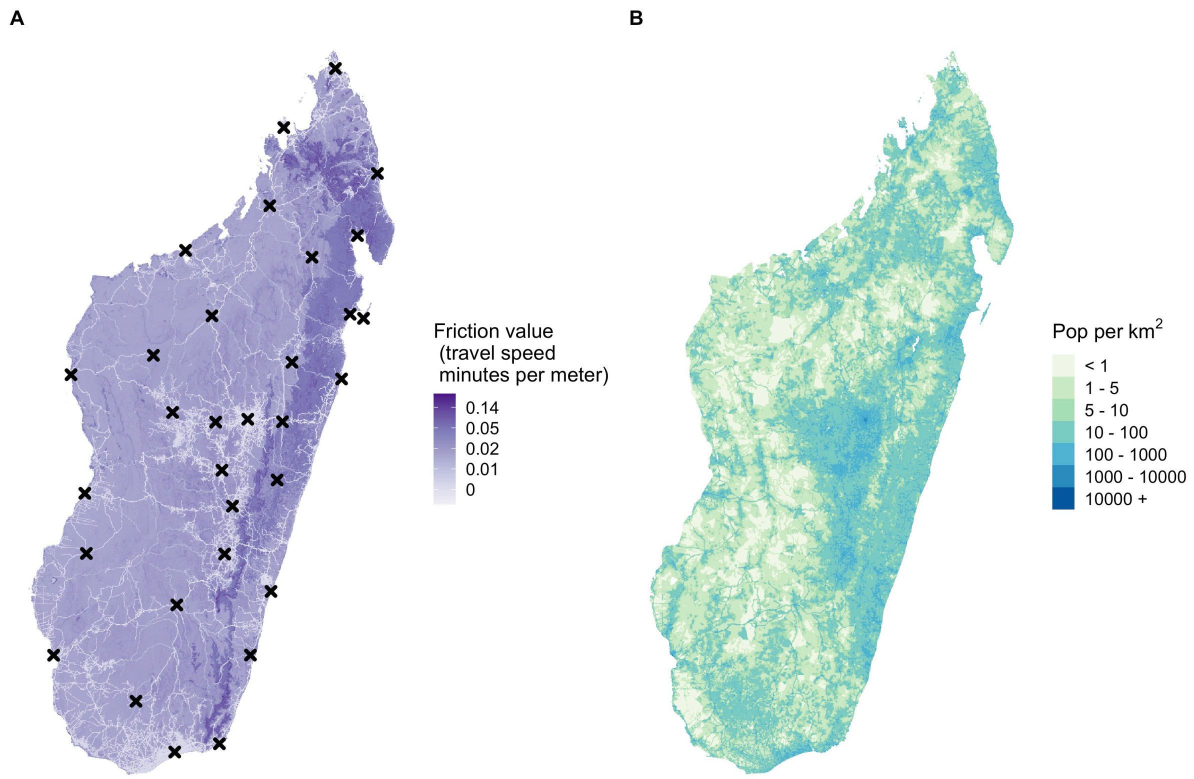

Fig A. Raster inputs to estimate travel times to the closest clinic provisioning PEP for Madagascar.

(A) Friction surface of travel speeds (in minutes per meter) at an ~ 1x1 km scale, with location of current clinics provisioning PEP (N = 31) shown with black x's (original source: Malaria Atlas Project friction surface, (https://malariaatlas.org/research-project/accessibility-to-cities/, CC-BY 3.0). (B) Population estimates resampled to the same friction surface (original source: WorldPop, https://www.worldpop.org/geodata/summary?id=70, CC-BY 4.0 license).

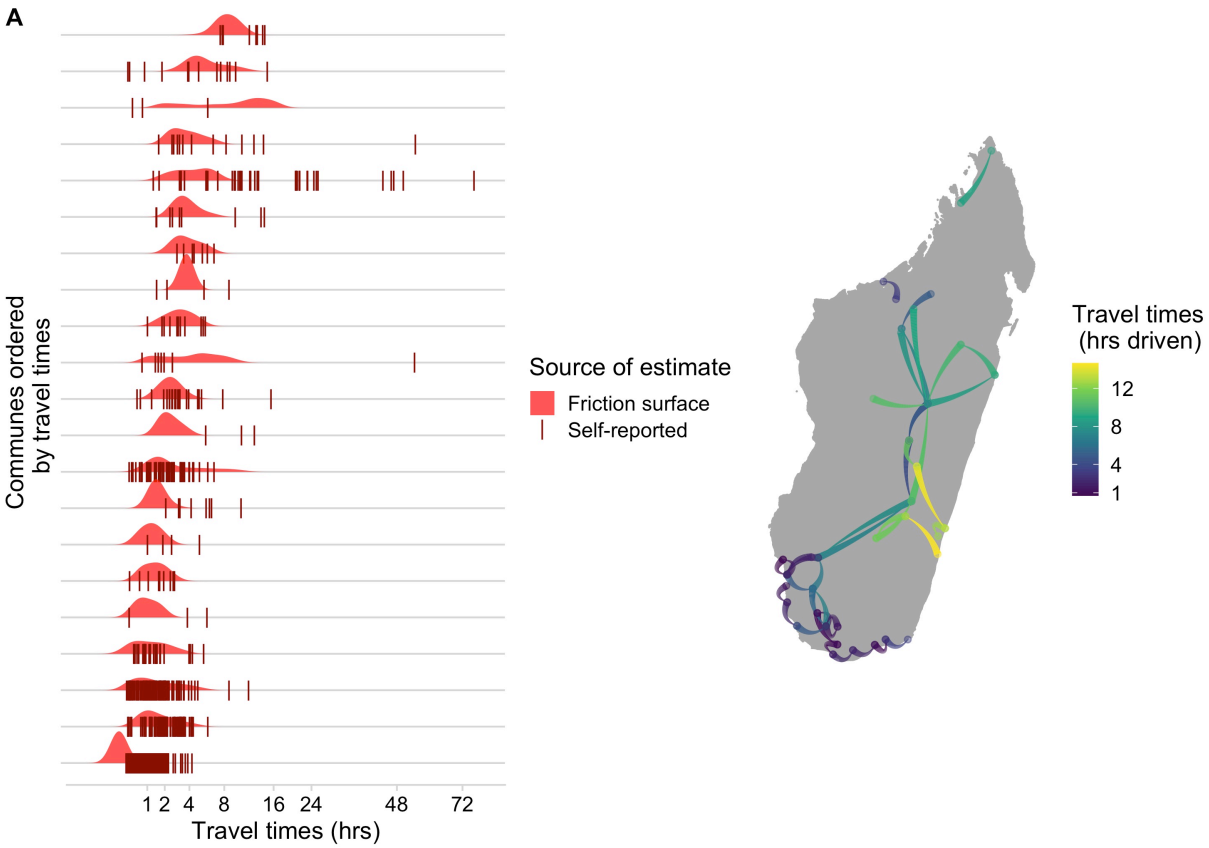

Fig B. Raw patient reported and driving travel time data.

(A) Distribution of travel times estimated at the grid cell level and reported by patients for each commune where patient data were available from the Moramanga PEP clinic (B) Reported driving times between locations, with the color corresponding to the total driving time and the size of the line showing the direction of travel (narrow to wide ~ origin to destination). Paths are Bezier curves from origin to destination, and do not show actual paths driven. Administrative boundaries from OCHA via HDX (https://data.humdata.org/dataset/madagascar-administrative-level-0-4-boundaries, CC-BY-IGO).

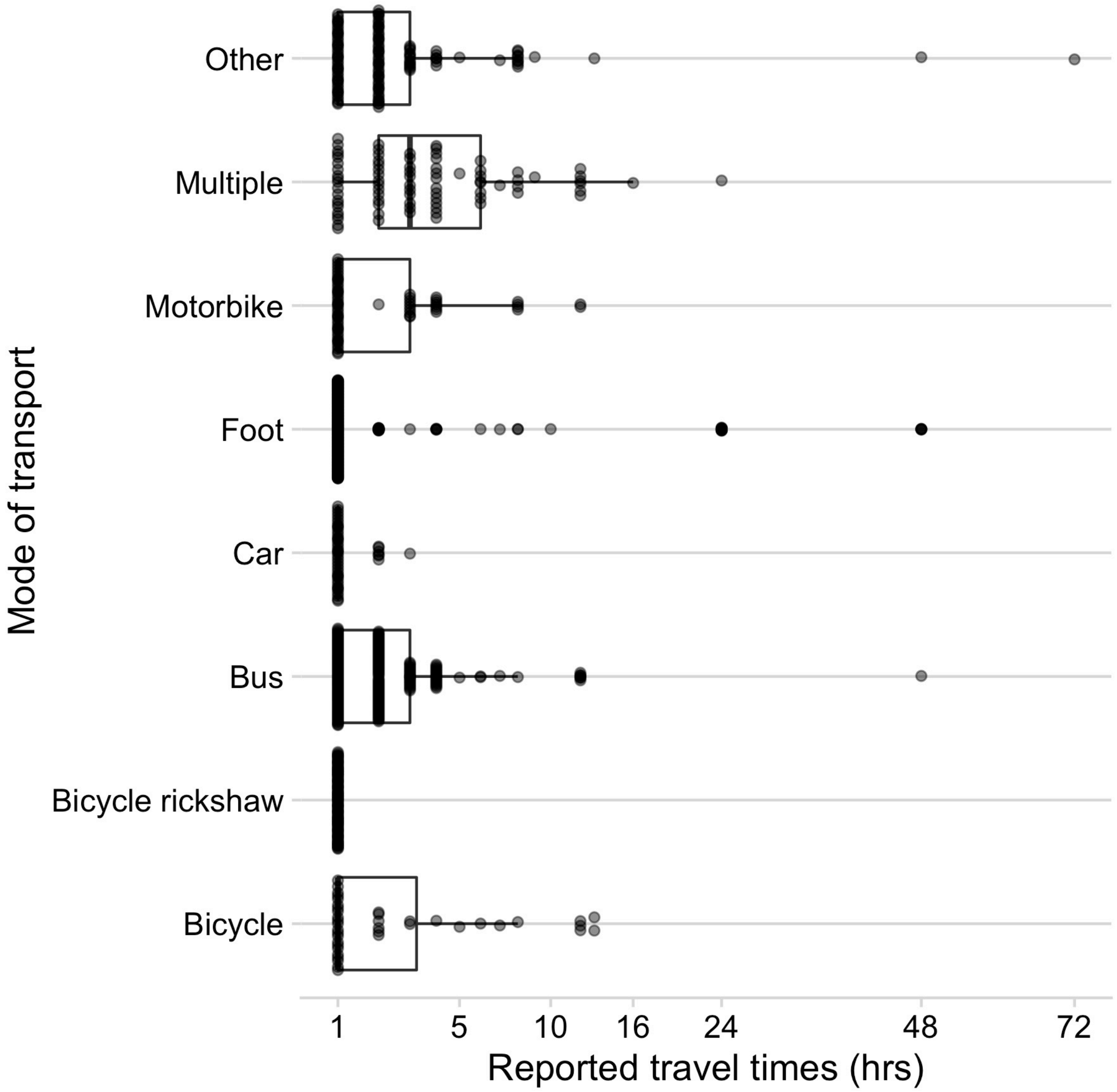

Fig C. Reported modes of transport used compared to reported travel times for patients reporting to the Moramanga PEP clinic

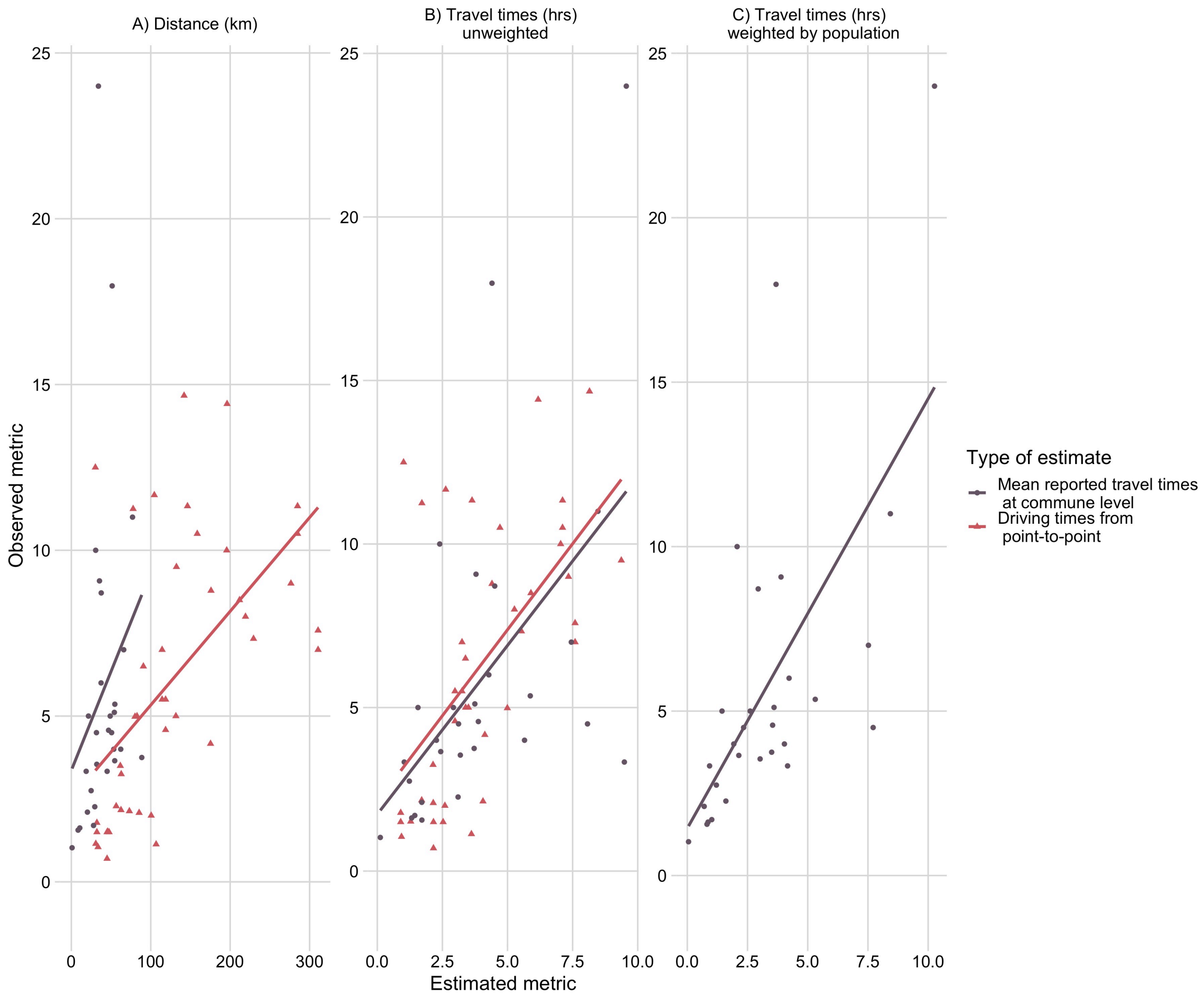

Fig D. Observed estimates of travel times (commune means of patient reported travel times and driving times between point locations) vs. estimates from friction surface.

Predicted by (A) Distance (km) (Euclidean distance between origin and destination for driving times and distance from the commune centroid to the Moramanga PEP clinic for commune means) (B) Travel time estimates and (C) Travel time estimates weighted by population (for commune means only). Administrative boundaries are from OCHA (https://data.humdata.org/dataset/madagascar-administrative-level-0-4-boundaries, CC-BY-IGO).

Table A. R^2 values from linear models with estimated access metrics predicting either driving times or commune means of patient reported travel times.

Predictor	Driving times	Commune mean of patient reported travel times
Weighted travel times (hrs)	NA	0.433
Unweighted travel times (hrs)	0.347	0.290
Distance (km)	0.368	0.093

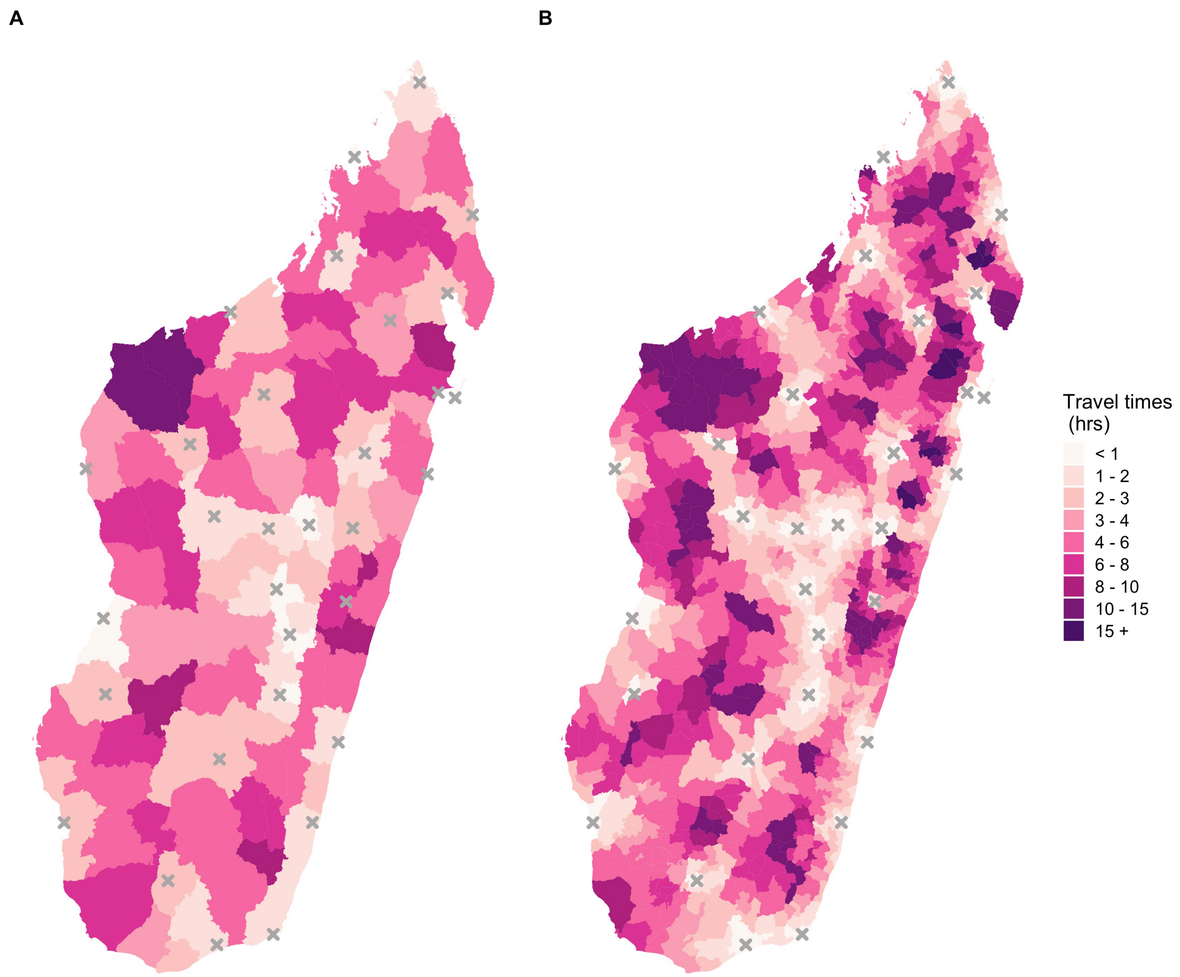

Fig E. Estimates of mean travel times weighted by population at the (A) District (B) Commune scale. Administrative boundaries from OCHA via HDX (https://data.humdata.org/dataset/madagascar-administrative-level-0-4-boundaries, CC-BY-IGO).
