# Supplementary material for: How geographic access to care shapes disease burden: The current impact of post-exposure prophylaxis and potential for expanded access to prevent human rabies deaths in Madagascar
Source: PLoS Negl Trop Dis. 2021 Apr 26;15(4):e0008821. doi: 10.1371/journal.pntd.0008821 (PMC8102000; doi:10.1371/journal.pntd.0008821)
Supplement: S2 Text — (A) Catchments as assigned by closest clinic for the majority of the population within a commune (polygon fill) or within a district (polygon outline). Admin units where the fill and border colors do not match show places where assigned catchments differ at the district vs commune scale. (B) Distribution of the proportion of the population in a given administrative unit (district or commune) served by the catchment assigned. (C) The proportion of bites reported to each clinic which originated from a district within the assigned catchment. The vertical line indicates the proportion of bites from within the assigned catchment for the Moramanga data (~90%). Administrative boundaries from OCHA via HDX (https://data.humdata.org/dataset/madagascar-administrative-level-0-4-boundaries, CC-BY-IGO). Fig B. Estimating under-submission of patient forms. (A) Number of patient forms submitted to IPM for each clinic over the study period for each clinic, with periods of time where no forms were submitted for > = 15 days excluded (in grey); (B) Estimates for the proportion of forms submitted for each clinic (points are the estimate for each year and the line is the range), calculated as the # of days in a year which were not excluded based on the criteria of 15 consecutive days of non-submission/365. (C) The difference between log(estimated) and log(observed) vials provisioned for the period of 2013–2017 for each clinic correcting for under-submission (squares) using the 15 day cut-off show in A and B, vs. not correcting for under-submission (circles). We did not have data on vials provisioned for IPM. Table A. Root mean squared error (MSE) between observed vials provisioned and estimated by the different consecutive day threshold for correcting for periods of no form submission, with the minimum root MSE in bold. (PDF) [file pntd.0008821.s002.pdf]

S2 Text. Estimating bite incidence

How geographic access to care shapes disease burden: the current impact of post-exposure prophylaxis and potential for expanded access to prevent human rabies deaths in Madagascar

Rajeev et al. 2021

Table of Contents

Fig A. Catchment assignments by travel time. 3
Fig B. Estimating under-submission of patient forms. 4
Table A. Root mean squared error (MSE) between observed vials provisioned and estimated by the different consecutive day threshold for correcting for periods of no form submission, with the minimum root MSE in bold. 5
References 5

Correspondence: Rajeev et al. 2021 <mrajeev@princeton.edu>

Most patients from each district reported to their closest clinic provisioning PEP by the weighted travel time metric (main text Fig 3). Accordingly, we assigned catchments based on which clinic was the closest for the majority of the population. While there are discrepancies between commune and district catchment assignments (Fig A.1), over 75% of the population in a given district or commune were closest to a single clinic (Fig A.3). We excluded any clinics which submitted less than 10 forms (excluded 11 catchments, main text Fig 3A grey polygons) and corrected for periods where clinics did not submit any forms.

Vial demand was simulated under simplified assumptions of PEP administration and adherence [1], based on patients reported randomly across the year. During this period, the Thai Red Cross Intradermal regimen was used across Madagascar, with 0.2 mL administered per patient completing doses on days 0, 3, 7, and 28. Vials can be shared within a day between two patients, resulting in 0.1ml wastage per vial shared, plus any

additional wastage from unused doses discarded at the end of the day. We estimate vial estimates as the midpoint estimate if all patients complete 3 vs. 4 doses. As clinic submission of forms was highly variable from 2014-2017 (Fig B.1), we compared estimated demand to the total vials provisioned across this four year period comparing different thresholds for correcting for periods of no form submission (i.e, designating periods of 1, 5, 10, 15, and 30 consecutive days with zero submitted records as missing, compared to no correction).

Estimates of vial demand based on uncorrected bite patient numbers were generally lower than the number of vials provisioned for those clinics with substantial under-submission of forms (Fig B.2). Correcting patient numbers for under-submission resulted in estimates of vial demand closer to the provisioned vials for most clinics (Fig B.3, Table A).

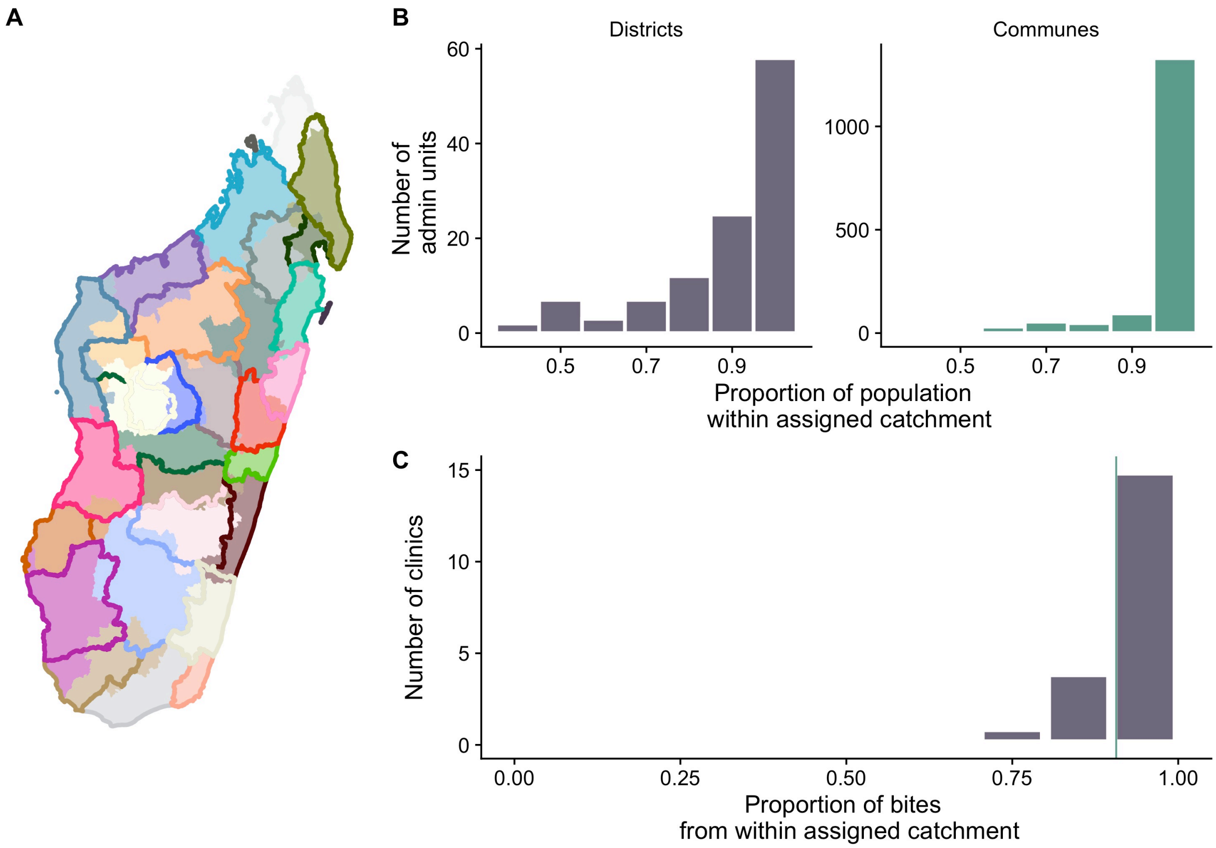

Fig A. Catchment assignments by travel time.

(A) Catchments as assigned by closest clinic for the majority of the population within a commune (polygon fill) or within a district (polygon outline). Admin units where the fill and border colors do not match show places where assigned catchments differ at the district vs commune scale. (B) Distribution of the proportion of the population in a given administrative unit (district or commune) served by the catchment assigned. (C) The proportion of bites reported to each clinic which originated from a district within the assigned catchment. The vertical line indicates the proportion of bites from within the assigned catchment for the Moramanga data (~90%). Administrative boundaries from OCHA via HDX (https://data.humdata.org/dataset/madagascar-administrative-level-0-4-boundaries, CC-BY-IGO).

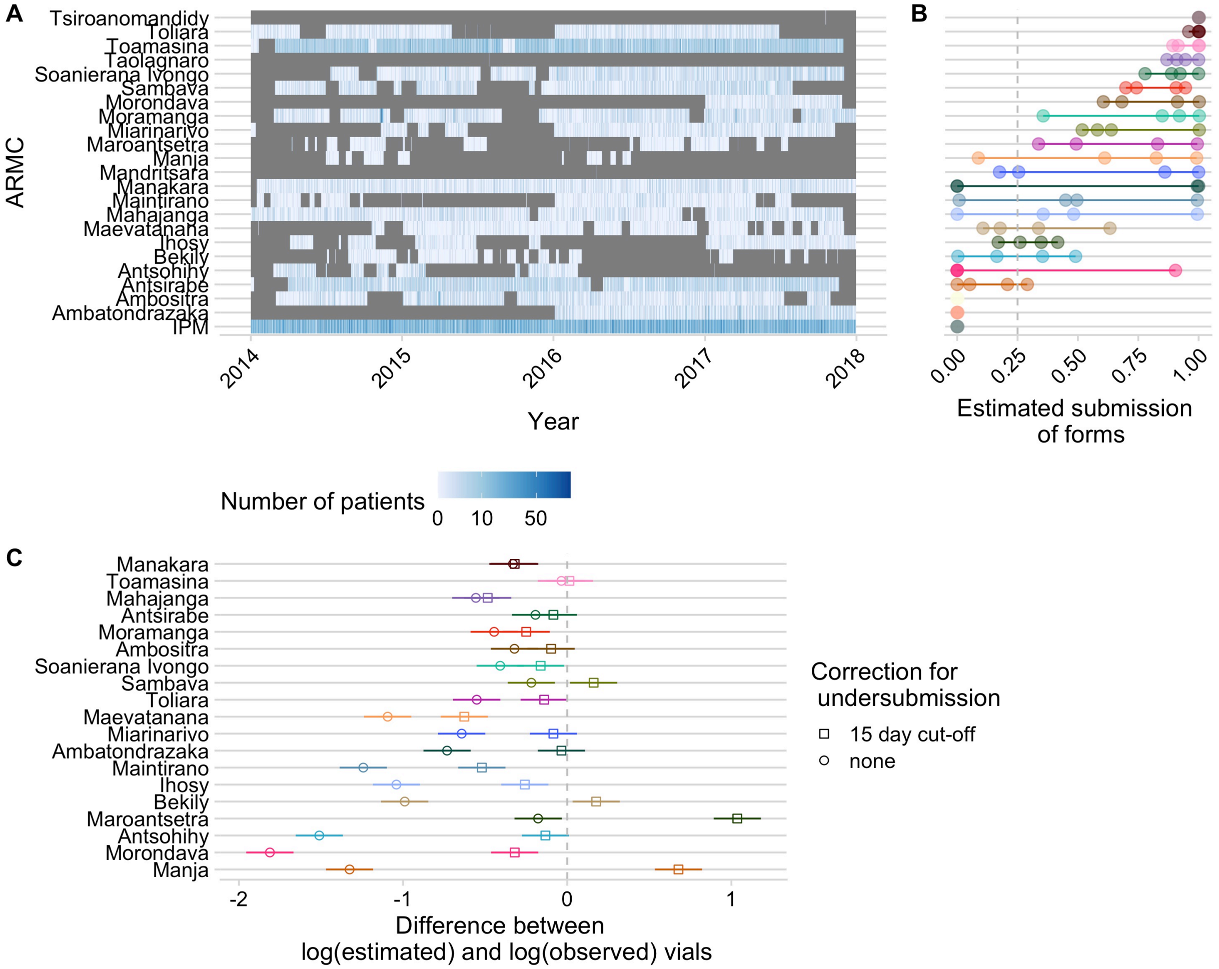

Fig B. Estimating under-submission of patient forms.

(A) Number of patient forms submitted to IPM for each clinic over the study period for each clinic, with periods of time where no forms were submitted for >= 15 days excluded (in grey); (B) Estimates for the proportion of forms submitted for each clinic (points are the estimate for each year and the line is the range), calculated as the # of days in a year which were not excluded based on the criteria of 15 consecutive days of non-submission/365. (C) The difference between log(estimated) and log(observed) vials provisioned for the period of 2013 - 2017 for each clinic correcting for under-submission (squares) using the 15 day cut-off show in A and B, vs. not correcting for under-submission (circles). We did not have data on vials provisioned for IPM.

Table A. Root mean squared error (MSE) between observed vials provisioned and estimated by the different consecutive day threshold for correcting for periods of no form submission, with the minimum root MSE in bold.

Consecutive day threshold	Root MSE
1	2346.38
5	1038.45
10	1015.88
**15**	**1006.82**
30	1063.39
No correction	1658.48

References

1. Rajeev M, Edosoa G, Hanitriniaina C, Andriamandimby SF, Guis H, Ramiandrasoa R, et al. Healthcare utilization, provisioning of post-exposure prophylaxis, and estimation of human rabies burden in madagascar. Vaccine. 2019;37: A35–A44.
