# Supplementary material for: How geographic access to care shapes disease burden: The current impact of post-exposure prophylaxis and potential for expanded access to prevent human rabies deaths in Madagascar
Source: PLoS Negl Trop Dis. 2021 Apr 26;15(4):e0008821. doi: 10.1371/journal.pntd.0008821 (PMC8102000; doi:10.1371/journal.pntd.0008821)
Supplement: S3 Text — For the column pop effect, addPop = models with population size as additional covariate, onlyPop = models with population as only covariate, flatPop = models with population as offset in model. For the intercept type: random = random intercept by catchment, fixed = a single fixed intercept was estimated). The Overdispersion column indicates whether an overdispersion parameter was estimated (yes) or not (no). Fig B. Prediction to data used to fit each model. Log of the observed bites against the log of predicted bites generated from sampling 1000 independent draws from the posterior distributions for each parameter, with the points the mean of the predictions and the linerange the 95% prediction intervals. Columns are by the type of model intercept (either a fixed intercept or a random intercept by catchment) and rows are the type of model structure with respect to the population covariate (addPop = population size as additional covariate, onlyPop = population as only covariate, flatPop = population as offset in model). Colors show which data set was used for fitting and the scale of the model (Moramanga = Moramanga data with covariates at the commune level, Commune = National data with covariates at the commune level, District = National data with covariates at the district level). Fig C. Out of fit predictions to data. Log of the observed bites against the log of predicted bites for data not used to fit the model. Predictions were generated by sampling 1000 independent draws from the posterior distributions for each parameter, with the points the mean of the predictions and the linerange the 95% prediction intervals. The first two columns are the predictions from the commune and district model fitted to the national data for the Moramanga data with fixed and random intercepts. The third column are predictions from models fitted to the Moramanga data for the national data at the commune and district scale (only fixed intercept models). Rows are the type of model str [file pntd.0008821.s003.pdf]

S3 Text. Modeling reported bite incidence

How geographic access to care shapes disease burden: the current impact of post-exposure prophylaxis and potential for expanded access to prevent human rabies deaths in Madagascar

Rajeev et al. 2021

Table of Contents

Fig A. Correlation between travel time in hours (the average weighted by the population) and population size of administrative units at the district and commune scale. 3
Table A. DIC and convergence estimates (maximum potential scale reduction factor and multivariate psrf, values < 1.1 indicate convergence) for all models. 4
Fig B. Prediction to data used to fit each model. 7
Fig C. Out of fit predictions to data. 8
Fig D. Posterior estimates of parameters from models with travel time and population as an offset. 9
Fig E. Predicted relationship between travel times (in hours) and reported bite incidence per 100,000 persons. 10
Fig F. Posterior estimates of the catchment intercepts (α parameters, with $\beta 0$ the estimated mean intercept) for models with and without an overdispersion parameter. 11
Fig G. Predicted relationship between travel times (in hours) and reported bite incidence per 100,000 persons. 12
Fig H. Posterior estimates for models with population as an offset and an overdispersion parameter. 13
Fig I. The estimated relationship between travel time in hours (x-axis) and mean annual bite incidence per 100,000 persons (y-axis). 14
References 15

Correspondence: Rajeev et al. 2021 <mrajeev@princeton.edu>

We used a weakly informative prior for the intercept of the models centered around the mean of the bite incidence for the given dataset with a standard deviation of 10. For the covariate terms, we centered the priors around zero with a standard deviation of 10. For

the variance terms (σ_0 and σ_e), we set uniform priors ($unif(0,5)$). We calculated the Deviance Information Criterion (DIC, a metric of model fit to data) for each candidate model as well as the maximum potential scale reduction factors (psrf) for each covariate and the multivariate psrf for the whole model (both are metrics of model convergence, where values < 1.1 are indicative of convergence, Table A) [1].

To test how well our models predicted the data, we sampled parameter estimates from the posterior distribution for each model to generate predictions to compare to data. In addition, we used the models to predict out-of-fit data (i.e. estimates from models fitted to the national data were used to predict the Moramanga data, and estimates from models fitted to the Moramanga data were used to predict the national data). Finally, to check how correcting for incomplete submission of forms affected our modeling results, we fitted our final models to the raw data uncorrected for submission (i.e. assuming forms were completely reported resulting in lower estimates of bite incidence) and with a lower cut-off (7 days, resulting in higher estimates of under-submission of forms).

For the national data, including a catchment random effect improved predictions (Fig B & Fig C). However, after accounting for overdispersion, catchment effects were not clearly identifiable (Table A) and the models resulted in similar predictions (Fig F & G), indicating that catchment effects could not be differentiated from random variation in the data. Similarly, while the commune model fit to the Moramanga data generated stronger travel time effects (Fig 4B), after accounting for data overdispersion, the posterior estimates of the parameters overlapped for the commune and district models fit to the national data (Fig D), and the model estimates were in general less robust to overdispersion than for the national data, particularly at low travel times (Fig E). Population size alone was the poorest fit to the data as estimated by DIC (Table A), and models with population size as an additional covariate did not generate realistic predictions to the observed data or when used to predict out of fit (Figs B and C).

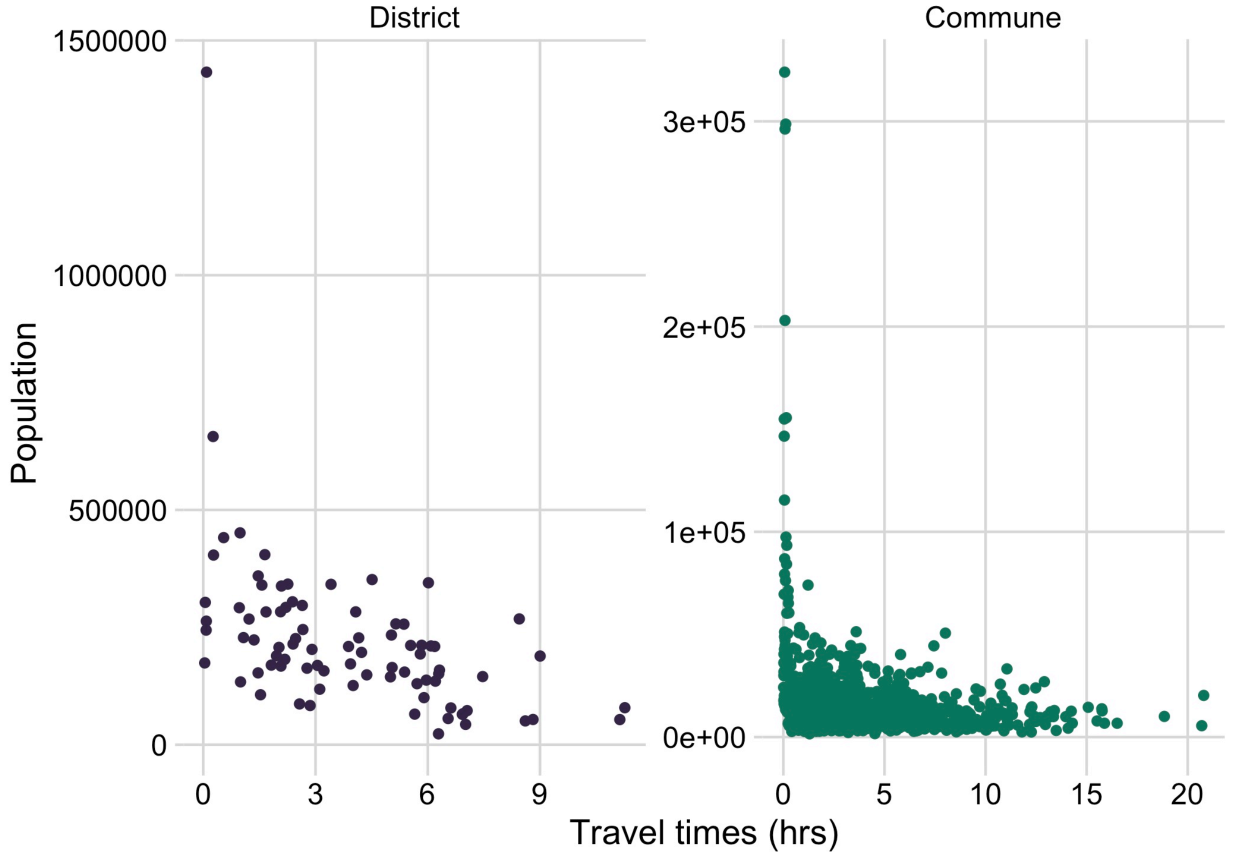

Fig A. Correlation between travel time in hours (the average weighted by the population) and population size of administrative units at the district and commune scale.

Table A. DIC and convergence estimates (maximum potential scale reduction factor and multivariate psrf, values < 1.1 indicate convergence) for all models. *For the column pop effect, addPop = models with population size as additional covariate, onlyPop = models with population as only covariate, flatPop = models with population as offset in model. For the intercept type: random = random intercept by catchment, fixed = a single fixed intercept was estimated). The Overdispersion column indicates whether an overdispersion parameter was estimated (yes) or not (no).*

Dataset	Scale	Pop effect	Intercept type	Overdispersion	DIC	Max psrf	Multivariate psrf
Moramanga	Commune	flatPop	fixed	no	10.664	1.001	1
Moramanga	Commune	onlyPop	fixed	no	12.122	1.001	1
**Moramanga**	**Commune**	**flatPop**	**fixed**	**yes**	**2.721**	**1.062**	**1.017**
Moramanga	Commune	addPop	fixed	no	8.425	1.011	1.003
National	Commune	addPop	fixed	no	119.917	1.002	1.001
National	Commune	onlyPop	random	no	144.383	1.001	1.001
National	Commune	onlyPop	fixed	no	213.415	1	1
National	Commune	flatPop	random	no	41.936	1.001	1.001
National	Commune	addPop	random	no	50.287	1.001	1.001
**National**	**Commune**	**flatPop**	**fixed**	**yes**	**6.784**	**1.028**	**1.008**
National	Commune	flatPop	random	yes	6.793	1.39	1.102

						3	
National	Commune	flatPop	fixed	no	89.813	1.001	1
National	District	flatPop	fixed	no	113.715	1.001	1
National	District	addPop	fixed	no	124.957	1.001	1
National	District	onlyPop	random	no	126.176	1.002	1.001
National	District	onlyPop	fixed	no	189.105	1.001	1
National	District	flatPop	random	no	59.12	1.001	1.001
**National**	**District**	**flatPop**	**fixed**	**yes**	**6.781**	**1.324**	**1.087**
National	District	flatPop	random	yes	6.783	1.122	1.069
National	District	addPop	random	no	61.133	1.004	1.001

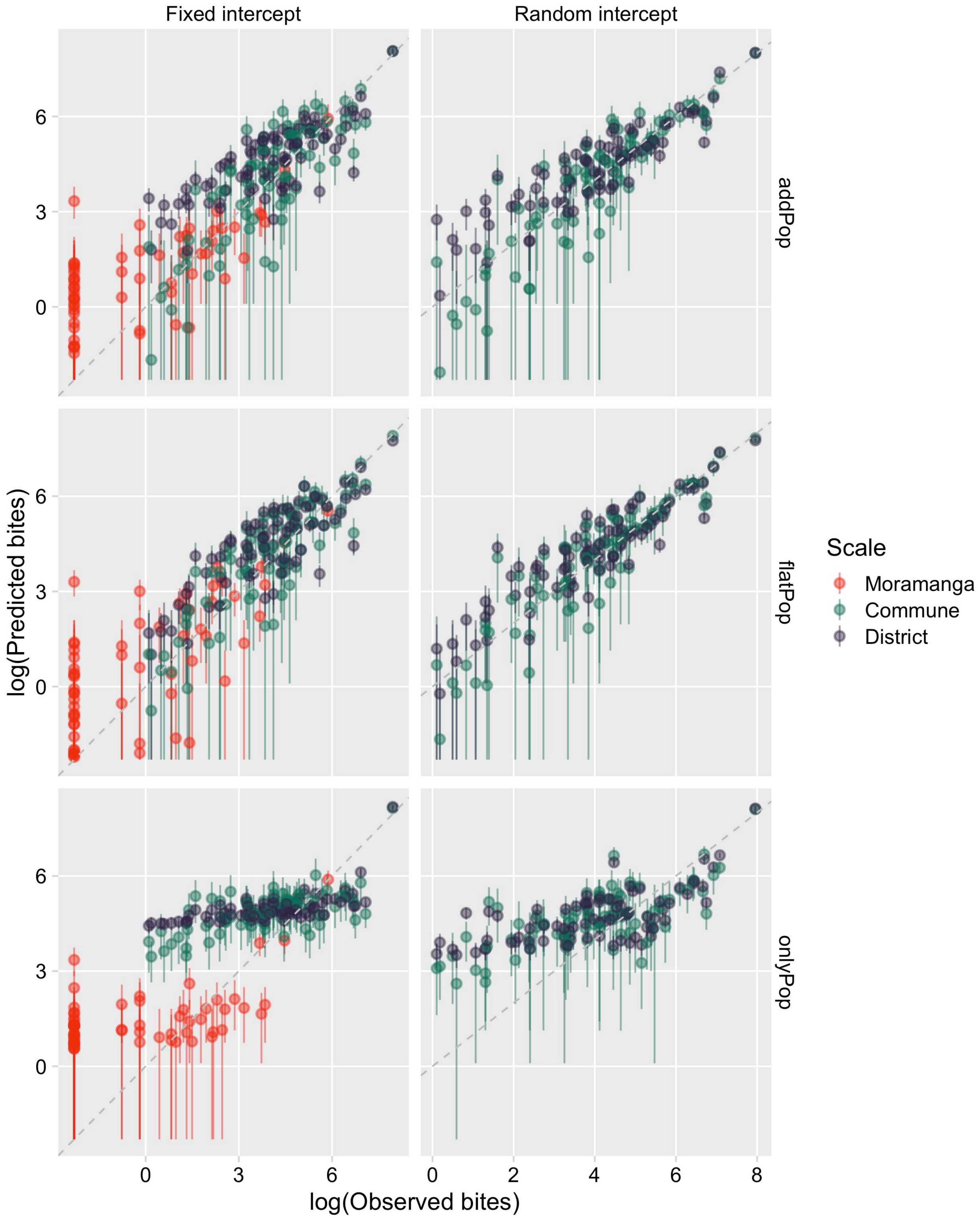

Fixed intercept
Random intercept
addPop
flatPop
onlyPop
log(Predicted bites)
log(Observed bites)
Scale
Moramanga
Commune
District
0
3
6
0
2
4
6
8

Fig B. Prediction to data used to fit each model.

Log of the observed bites against the log of predicted bites generated from sampling 1000 independent draws from the posterior distributions for each parameter, with the points the mean of the predictions and the linerange the 95% prediction intervals. Columns are by the type of model intercept (either a fixed intercept or a random intercept by catchment) and rows are the type of model structure with respect to the population covariate (addPop = population size as additional covariate, onlyPop = population as only covariate, flatPop = population as offset in model). Colors show which data set was used for fitting and the scale of the model (Moramanga = Moramanga data with covariates at the commune level, Commune = National data with covariates at the commune level, District = National data with covariates at the district level).

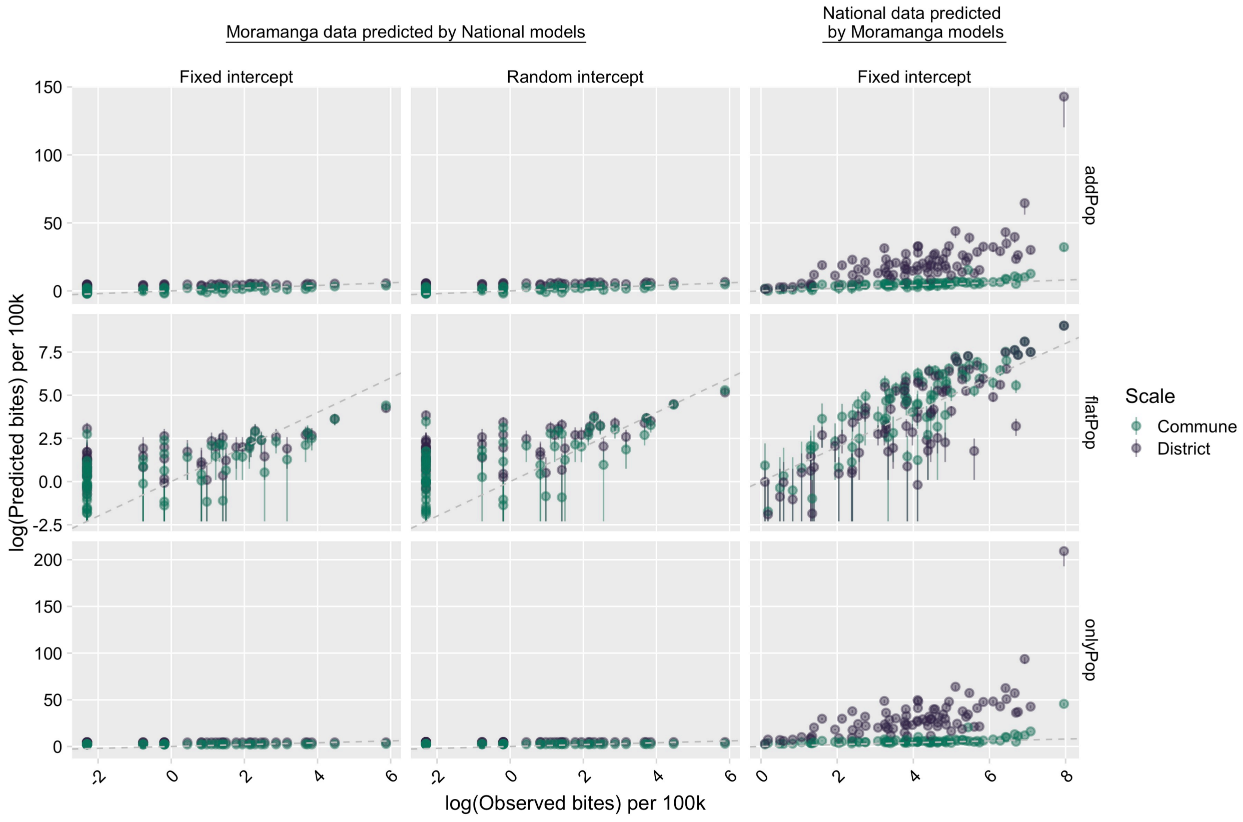

Fig C. Out of fit predictions to data.

Log of the observed bites against the log of predicted bites for data not used to fit the model. Predictions were generated by sampling 1000 independent draws from the posterior distributions for each parameter, with the points the mean of the predictions and the linerange the 95% prediction intervals. The first two columns are the predictions from the commune and district model fitted to the national data for the Moramanga data with fixed and random intercepts. The third column are predictions from models fitted to the Moramanga data for the national data at the commune and district scale (only fixed intercept models). Rows are the type of model structure with respect to the population covariate and colors show which data set was used for fitting as per Fig S3.2.

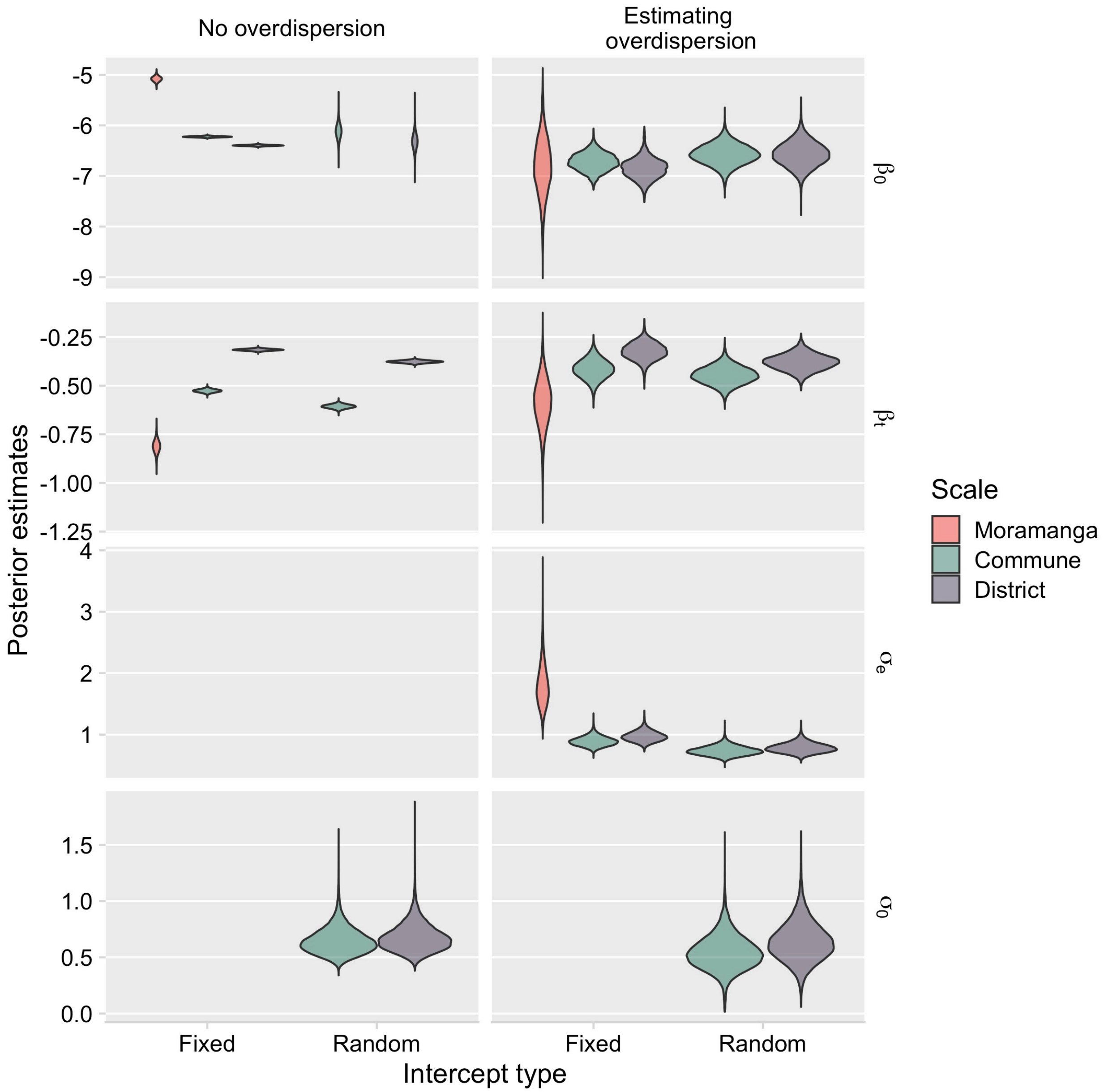

Fig D. Posterior estimates of parameters from models with travel time and population as an offset.

Comparing models accounting for overdispersion (σ_e) compared to models with no overdispersion parameter (flatPop in Figs S3.2 & S3.3). For the Moramanga model, as data came from a single catchment, models with a random catchment effect (σ_0) were not fitted.

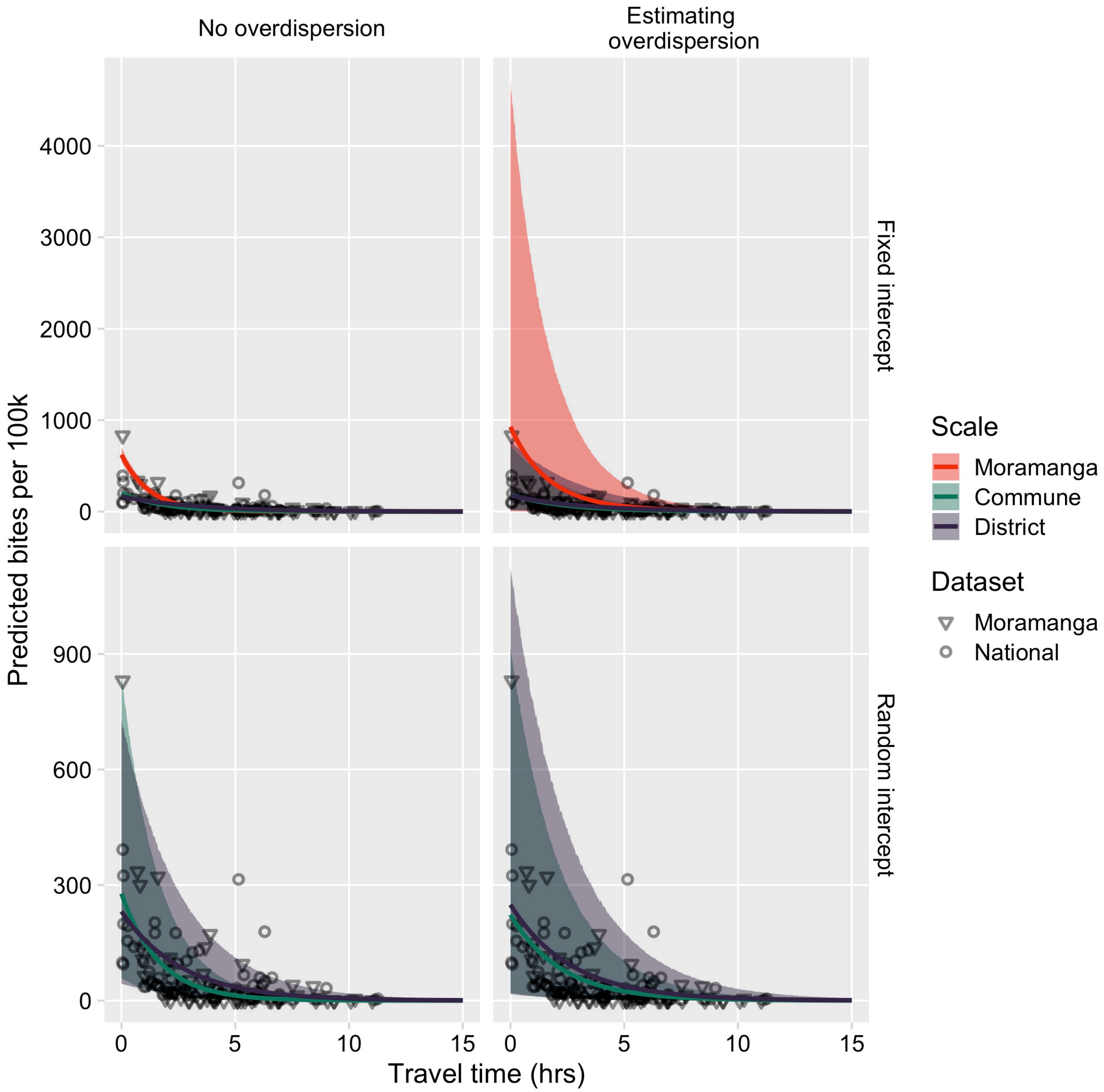

Fig E. Predicted relationship between travel times (in hours) and reported bite incidence per 100,000 persons.

Generated from sampling 1000 independent draws from the posterior distributions for each parameter, with the line the mean of the predictions and the envelopes showing the 95% prediction intervals. Rows are by the type of model intercept (either a fixed intercept or a random intercept by catchment) and columns are whether the model estimated an overdispersion parameter. The points show the data used to fit the models

(the National dataset), as well as the Moramanga dataset. Note the different y-axis limits between the fixed and random intercept models.

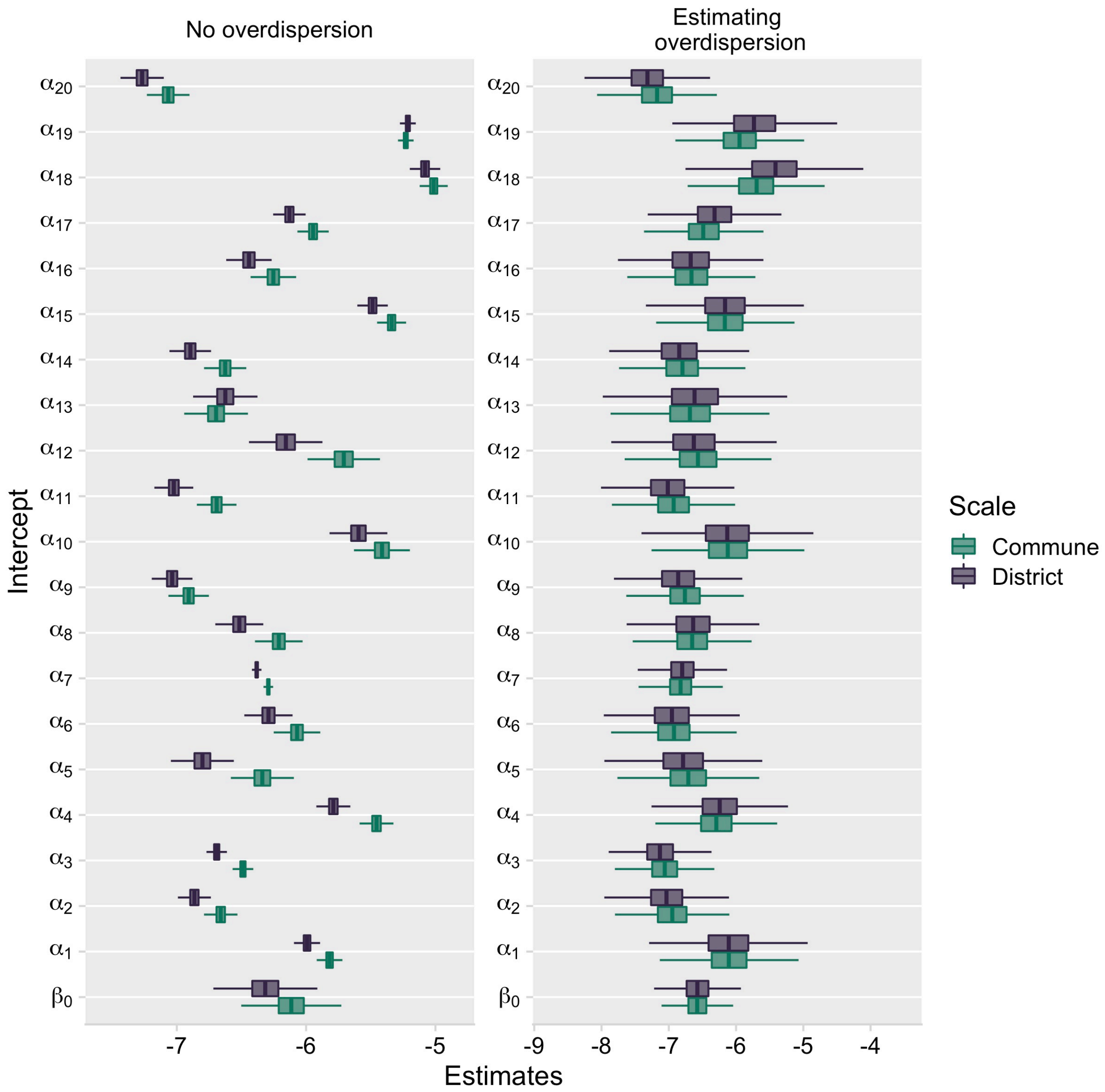

Fig F. Posterior estimates of the catchment intercepts (α parameters, with β_0 the estimated mean intercept) for models with and without an overdispersion parameter.

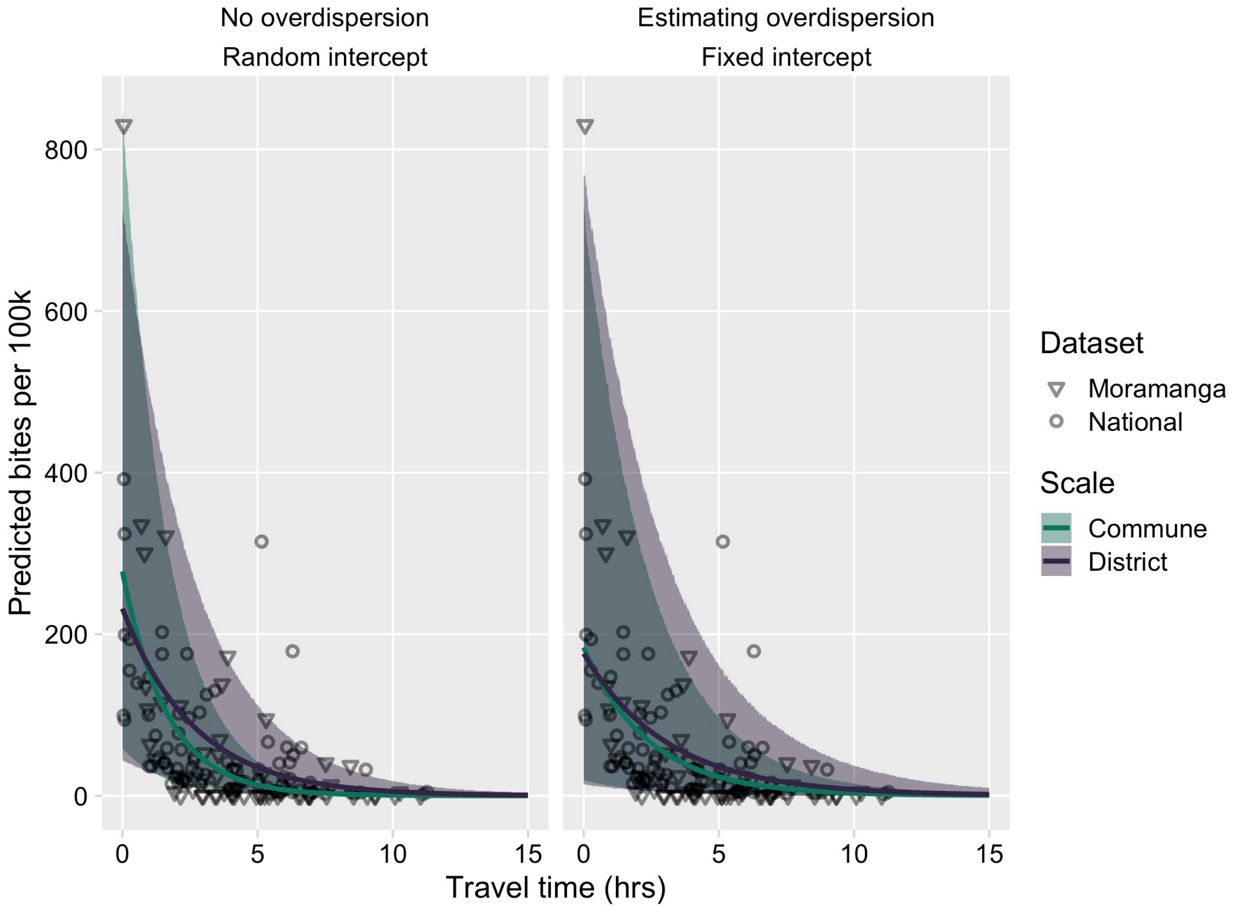

Fig G. Predicted relationship between travel times (in hours) and reported bite incidence per 100,000 persons.

For random intercept model without overdispersion vs. fixed intercept model with overdispersion, generated from sampling 1000 independent draws from the posterior distributions for each parameter, with the line the mean of the predictions and the envelopes showing the 95% prediction intervals. The points show the data used to fit the models (the National dataset), as well as the Moramanga dataset.

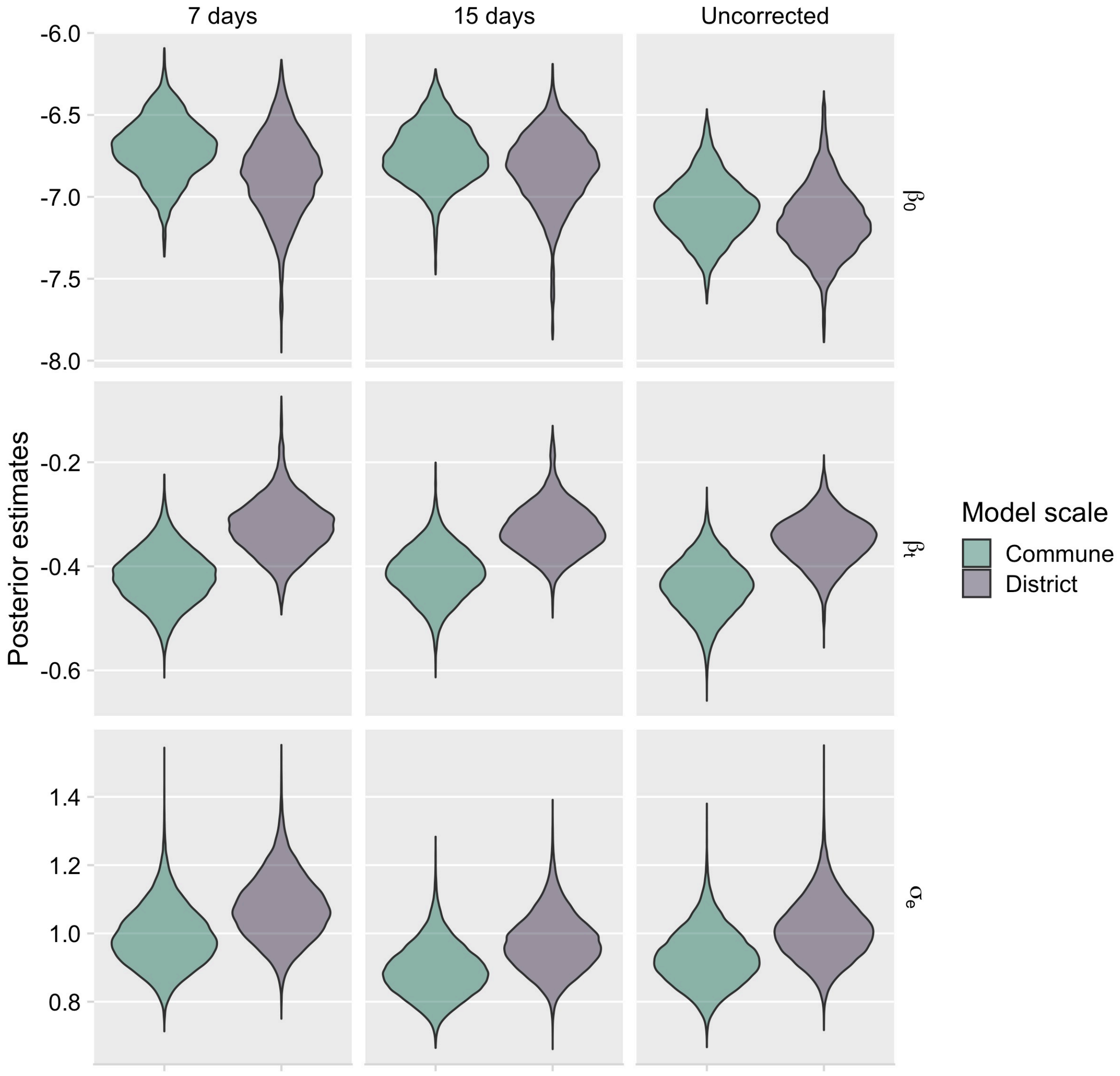

Fig H. Posterior estimates for models with population as an offset and an overdispersion parameter.

Columns show estimates from models fitted to the national dataset (1) corrected for both under-submission by correcting for periods of at least 7 days where zero patient forms were submitted, (2) correcting for periods of 15 days where zero patient forms were submitted, and (3) with the raw data not correcting for under-submission.

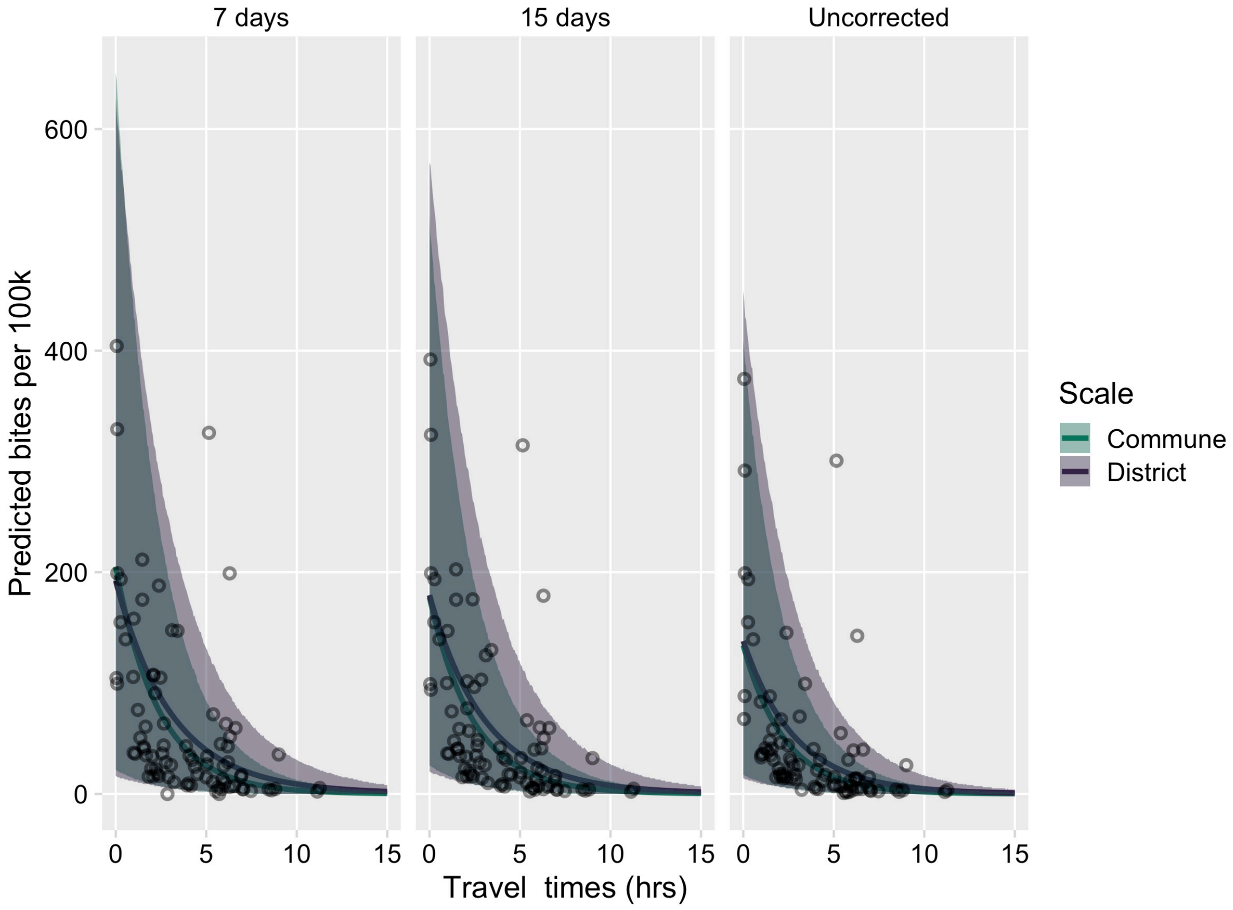

Fig I. The estimated relationship between travel time in hours (x-axis) and mean annual bite incidence per 100,000 persons (y-axis).

For models with population as an offset and an overdispersion parameter. Panels show predictions from models fitted to the national dataset 1) correcting for periods of at least 7 days where zero patient forms were submitted (a less stringent cutoff resulting in lower estimates of the proportion of forms submitted and thus higher estimates of reported bite incidence), (2) correcting for periods of 15 days where zero patient forms where submitted, as presented in the main analysis, and (3) with the raw data not correcting for under-submission (resulting in lower estimates of reported bite incidence). Predictions were generated from sampling 1000 independent draws from the posterior distributions for each parameter, with the line the mean of the predictions and the envelopes showing the 95% prediction intervals. The points show the data used to fit the models.

References

1. Gelman A, Hill J. Data analysis using regression and multilevel/hierarchical models. Cambridge university press; 2006.
