# Supplementary material for: How geographic access to care shapes disease burden: The current impact of post-exposure prophylaxis and potential for expanded access to prevent human rabies deaths in Madagascar
Source: PLoS Negl Trop Dis. 2021 Apr 26;15(4):e0008821. doi: 10.1371/journal.pntd.0008821 (PMC8102000; doi:10.1371/journal.pntd.0008821)
Supplement: S4 Text — Assuming that each dog on average exposes 0.39 persons. The black dashed lines show the range of human to dog ratios (HDRs) we use in the main analysis to estimate the range of human exposure incidence (where the red horizontal line and black dashed lines intersect). The grey dashed lines show the HDRs estimated from the Moramanga district from a recent study. The cells with red outlines show the range of estimated exposure incidence from a previous study of bite patients in Moramanga District. Administrative boundaries from OCHA via HDX (https://data.humdata.org/dataset/madagascar-administrative-level-0-4-boundaries, CC-BY-IGO). Fig B. Range of constrained scaling factors generated for district and commune population size. Underlying rabies exposures either (A) decreases with increasing population size or (B) increases with increasing population size across a fixed range of exposure incidence (15.6–76 exposures/100k persons). Lines show the expected relationship, with points showing where administrative units fall along this curve, and maps show how this results in variation in assumed exposure incidence spatially at the commune and district level. (PDF) [file pntd.0008821.s004.pdf]

S4 Text. Range of rabies exposure incidence in people

How geographic access to care shapes disease burden: the current impact of post-exposure prophylaxis and potential for expanded access to prevent human rabies deaths in Madagascar

Rajeev et al. 2021

Table of Contents

Fig A. Estimated exposures per 100,000 given a range of human to dog ratios (HDRs, x-axis) and annual dog rabies incidence (y axis). 2
Fig B. Range of constrained scaling factors generated for district and commune population size.......... 4
References.......... 4

Correspondence: Rajeev et al. 2021 <mrajeev@princeton.edu>

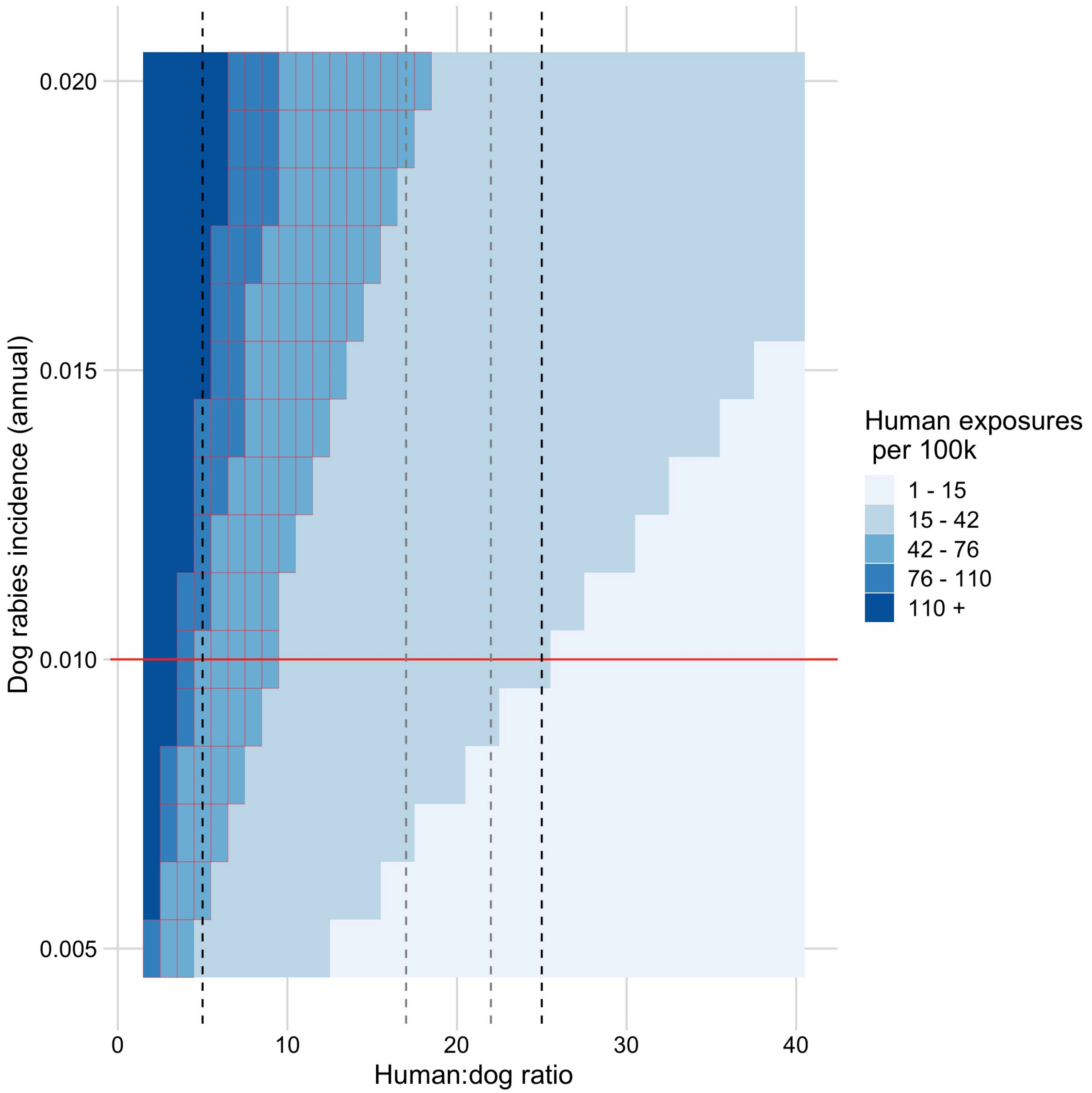

Fig A. Estimated exposures per 100,000 given a range of human to dog ratios (HDRs, x-axis) and annual dog rabies incidence (y axis).

Assuming that each dog on average exposes 0.39 persons [1]. The black dashed lines show the range of human to dog ratios (HDRs) we use in the main analysis to estimate the range of human exposure incidence (where the red horizontal line and black dashed lines intersect). The grey dashed lines show the HDRs estimated from the Moramanga district from a recent study [2]. The cells with red outlines show the range of estimated exposure incidence from a previous study of bite patients in Moramanga District [3].

Administrative boundaries from OCHA via HDX (https://data.humdata.org/dataset/madagascar-administrative-level-0-4-boundaries, CC-BY-IGO).

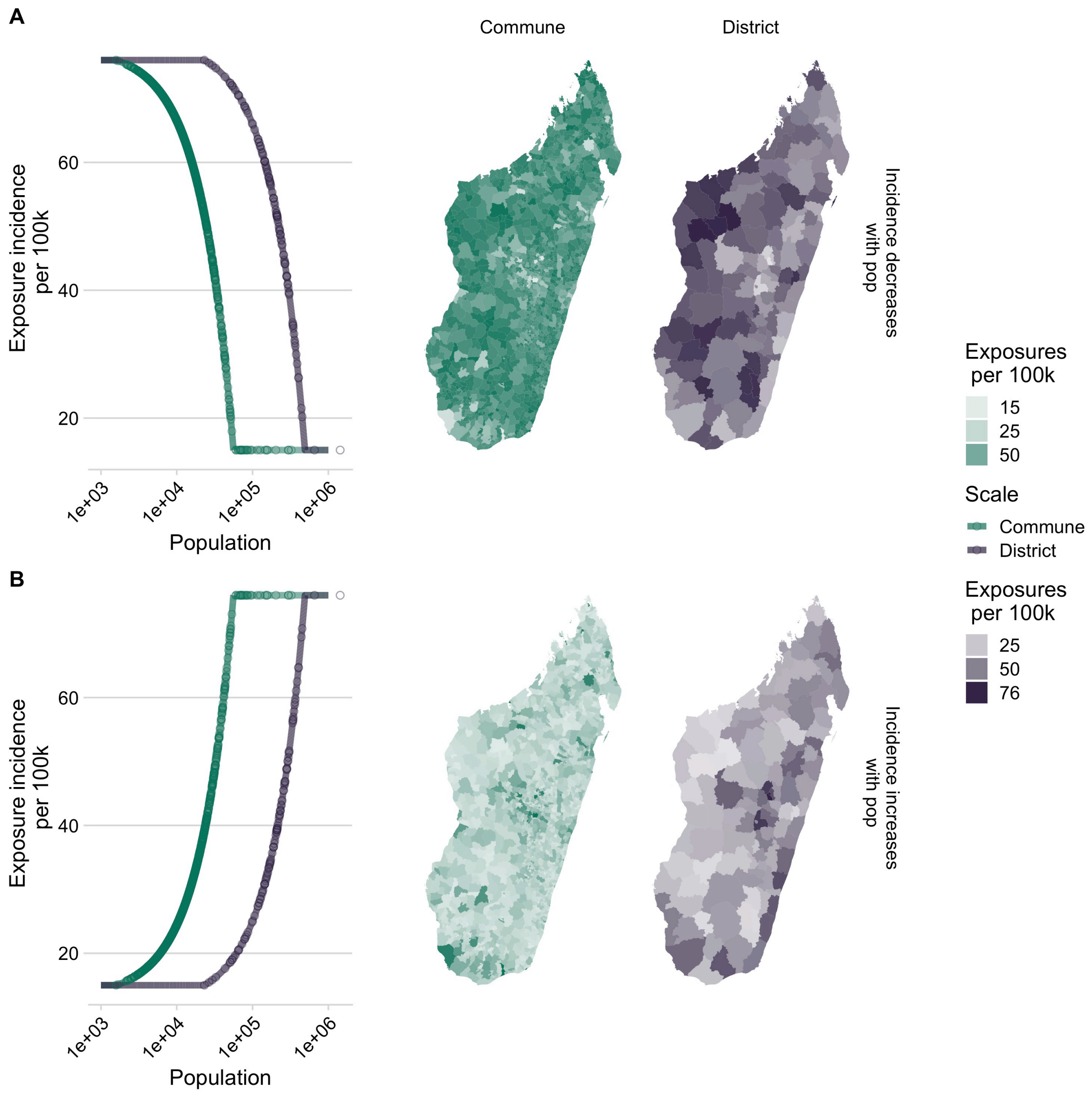

Fig B. Range of constrained scaling factors generated for district and commune population size

Underlying rabies exposures either (A) decreases with increasing population size or (B) increases with increasing population size across a fixed range of exposure incidence (15.6 - 76 exposures/100k persons). Lines show the expected relationship, with points showing where administrative units fall along this curve, and maps show how this results in variation in assumed exposure incidence spatially at the commune and district level.

References

1. Hampson K, Abela-Ridder B, Brunker K, Bucheli STM, Carvalho M, Caldas E, et al. Surveillance to Establish Elimination of Transmission and Freedom from Dog-mediated Rabies. bioRxiv. 2016.

2. Leblanc C. Rabies in Madagascar: A three-pronged survey of knowledge and practices among health care providers from anti-rabies treatment centers, veterinarians and the community of Moramanga. PhD thesis, Cnam de Sante Publique. 2019.

3. Rajeev M, Edosoa G, Hanitriniaina C, Andriamandimby SF, Guis H, Ramiandrasoa R, et al. Healthcare utilization, provisioning of post-exposure prophylaxis, and estimation of human rabies burden in madagascar. Vaccine. 2019;37: A35–A44.
