# Supplementary material for: How geographic access to care shapes disease burden: The current impact of post-exposure prophylaxis and potential for expanded access to prevent human rabies deaths in Madagascar
Source: PLoS Negl Trop Dis. 2021 Apr 26;15(4):e0008821. doi: 10.1371/journal.pntd.0008821 (PMC8102000; doi:10.1371/journal.pntd.0008821)
Supplement: S5 Text — We simulated expansion using three different ranking metrics: 1) reduction in mean travel times (green line) 2) the proportion of the population for which travel times were reduced (red dashed line) and 3) the proportion of the population for which travel times were reduced weighted by the reduction in travel times (pink dashed line). For each of these, we simulated burden using our decision tree framework (y axis is the mean of 1000 simulations of annual deaths at the national level). The blue lines show 10 simulations of randomly expanding access on reducing burden as a comparator. The panels show to the commune and district model of reported bite incidence. Fig B. Map of the location and at what step each clinic was added. The circles are each of the primary clinics across the country sized by the resulting average reduction in burden (based on smoothed annual burden estimates from the commune model, see inset). The large white crosses show the location of the existing 31 clinics provisioning PEP in Madagascar and the smaller white crosses are the additional primary clinics in the country which were added in the final step but not ranked. Administrative boundaries from OCHA via HDX (https://data.humdata.org/dataset/madagascar-administrative-level-0-4-boundaries, CC-BY-IGO). Fig C. Maps of how travel times change as clinics are added. (A) at the ~1 x 1 km grid cell (B) commune and (C) district scales. The columns are ordered by the number or clinics at each step: baseline (N = 31), + 83 (1 per district), + 200, + 600, + 1406 (1 per commune), and max (+ 1696 clinics, all additional primary clinics in the country). Grey pixels show the location of clinics provisioning PEP at each step. Commune and district values are the average grid cell travel times weighted by the population in each cell. Administrative boundaries from OCHA via HDX (https://data.humdata.org/dataset/madagascar-administrative-level-0-4-boundaries, CC-BY-IGO). Fig D. Shifts in key metrics as clinics [file pntd.0008821.s005.pdf]

S5 Text. Estimating the impact of expanding PEP provisioning to additional clinics

How geographic access to care shapes disease burden: the current impact of post-exposure prophylaxis and potential for expanded access to prevent human rabies deaths in Madagascar

Rajeev et al. 2021

Table of Contents

Fig A. Comparing metrics for ranking clinics for targeted expansion. 2
Fig B. Map of the location and at what step each clinic was added. 3
Fig C. Maps of how travel times change as clinics are added. 4
Fig D. Shifts in key metrics as clinics are added. 5
Fig E. Shifts in key metrics as clinics are added. 6
Fig F. Shifts in key metrics as clinics are added. 7
Fig G. Shifts in where bites are reported to as clinics are added for the commune model. 8
Fig H. Shifts in where bites are reported to as clinics are added for the district model. 9

Correspondence: Rajeev et al. 2021 <mrajeev@princeton.edu>

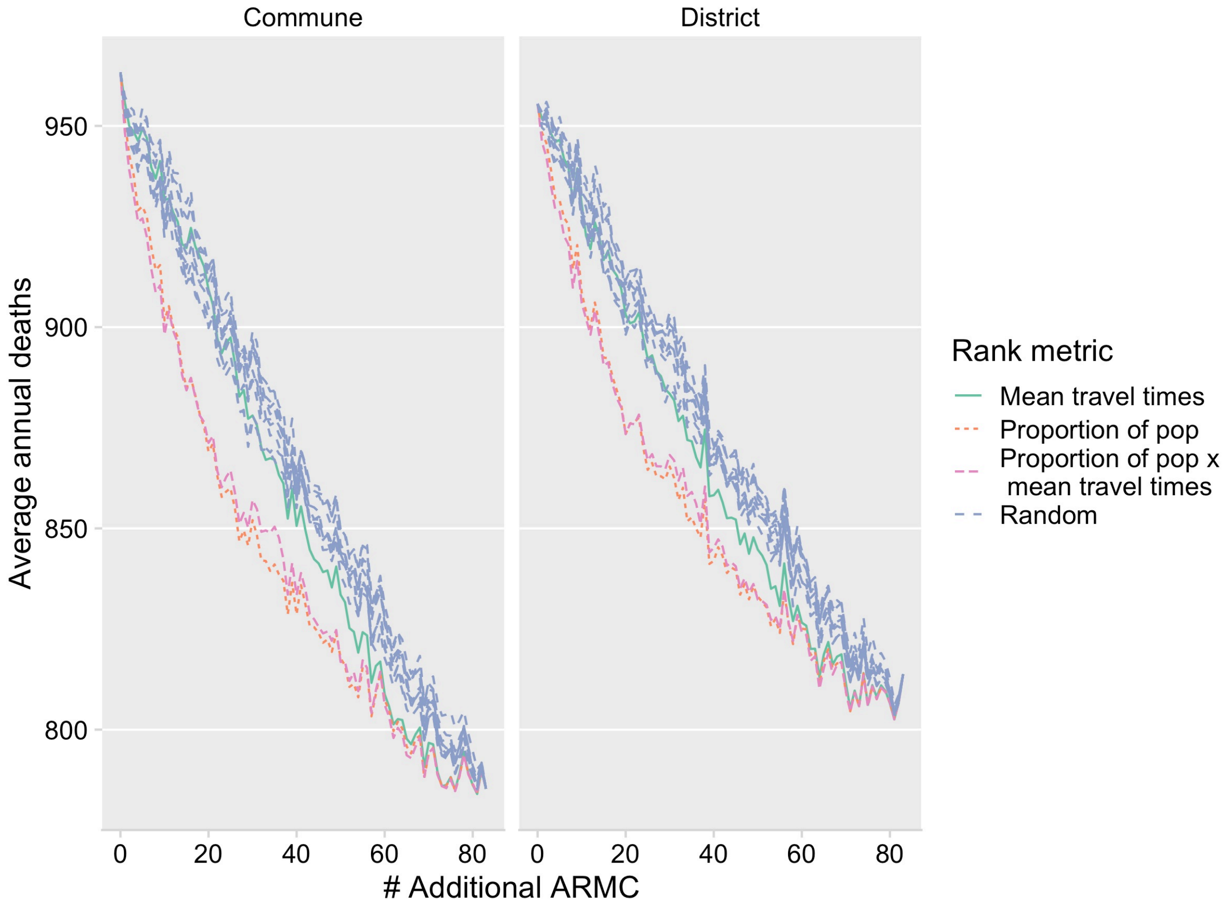

Fig A. Comparing metrics for ranking clinics for targeted expansion.

We simulated expansion using three different ranking metrics: 1) reduction in mean travel times (green line) 2) the proportion of the population for which travel times were reduced (red dashed line) and 3) the proportion of the population for which travel times were reduced weighted by the reduction in travel times (pink dashed line). For each of these, we simulated burden using our decision tree framework (y axis is the mean of 1000 simulations of annual deaths at the national level). The blue lines show 10 simulations of randomly expanding access on reducing burden as a comparator. The panels show to the commune and district model of reported bite incidence.

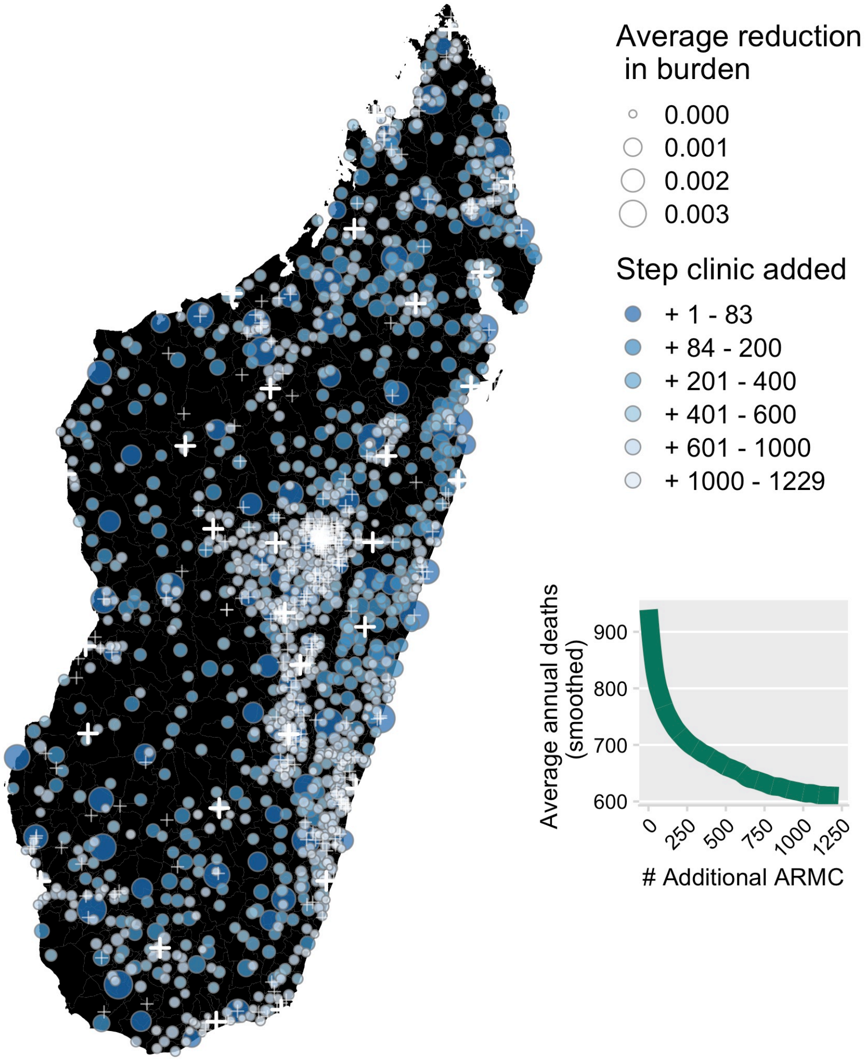

Fig B. Map of the location and at what step each clinic was added.

The circles are each of the primary clinics across the country sized by the resulting average reduction in burden (based on smoothed annual burden estimates from the commune model, see inset). The large white crosses show the location of the existing 31 clinics provisioning PEP in Madagascar and the smaller white crosses are the additional primary clinics in the country which were added in the final step but not ranked. Administrative boundaries from OCHA via HDX

(https://data.humdata.org/dataset/madagascar-administrative-level-0-4-boundaries, CC-BY-IGO).

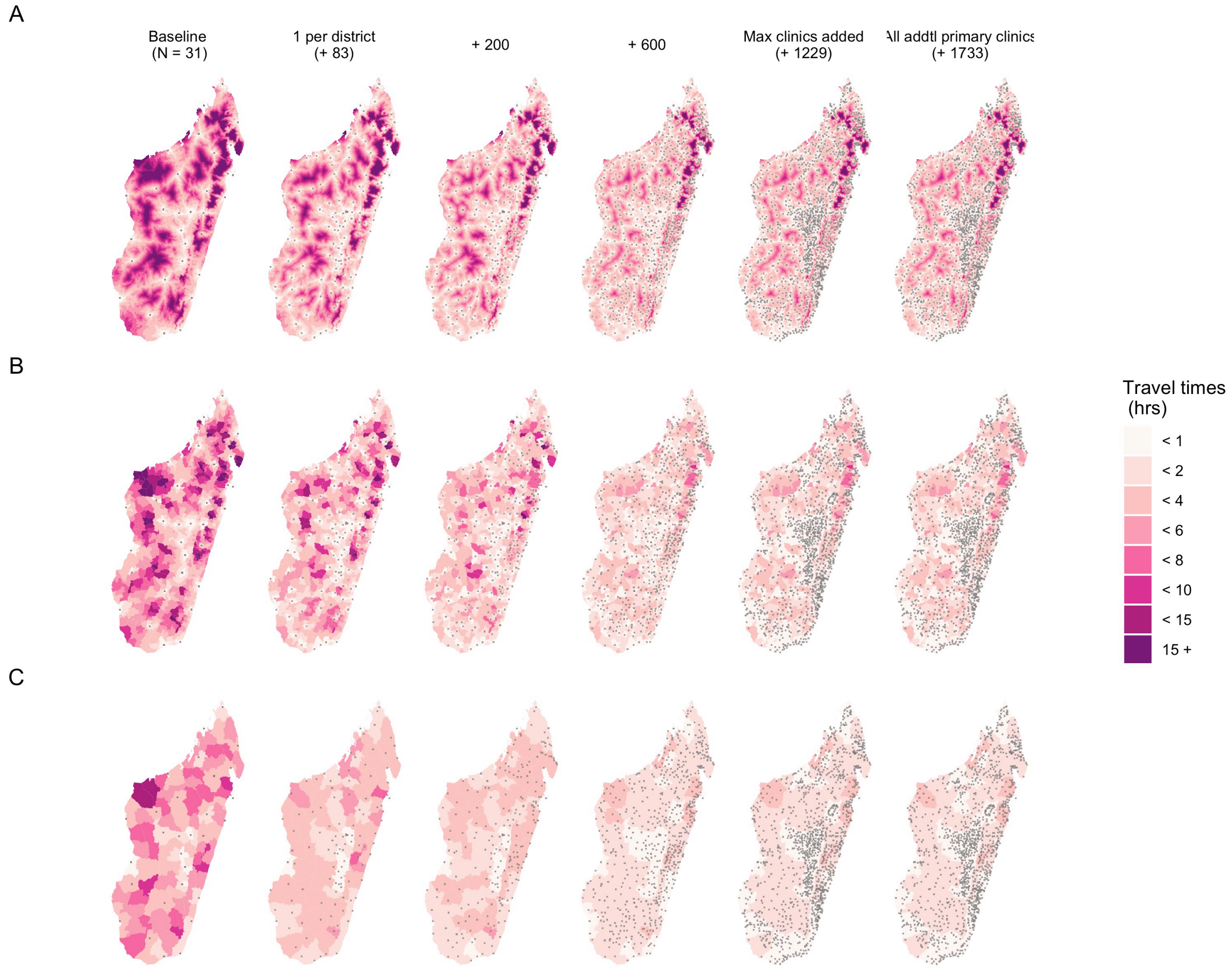

Fig C. Maps of how travel times change as clinics are added.

(A) at the ~1 x 1 km grid cell (B) commune and (C) district scales. The columns are ordered by the number or clinics at each step: baseline (N = 31), + 83 (1 per district), + 200, + 600, + 1406 (1 per commune), and max (+ 1696 clinics, all additional primary clinics in the country). Grey pixels show the location of clinics provisioning PEP at each step. Commune and district values are the average grid cell travel times weighted by the population in each cell. Administrative boundaries from OCHA via HDX (https://data.humdata.org/dataset/madagascar-administrative-level-0-4-boundaries, CC-BY-IGO).

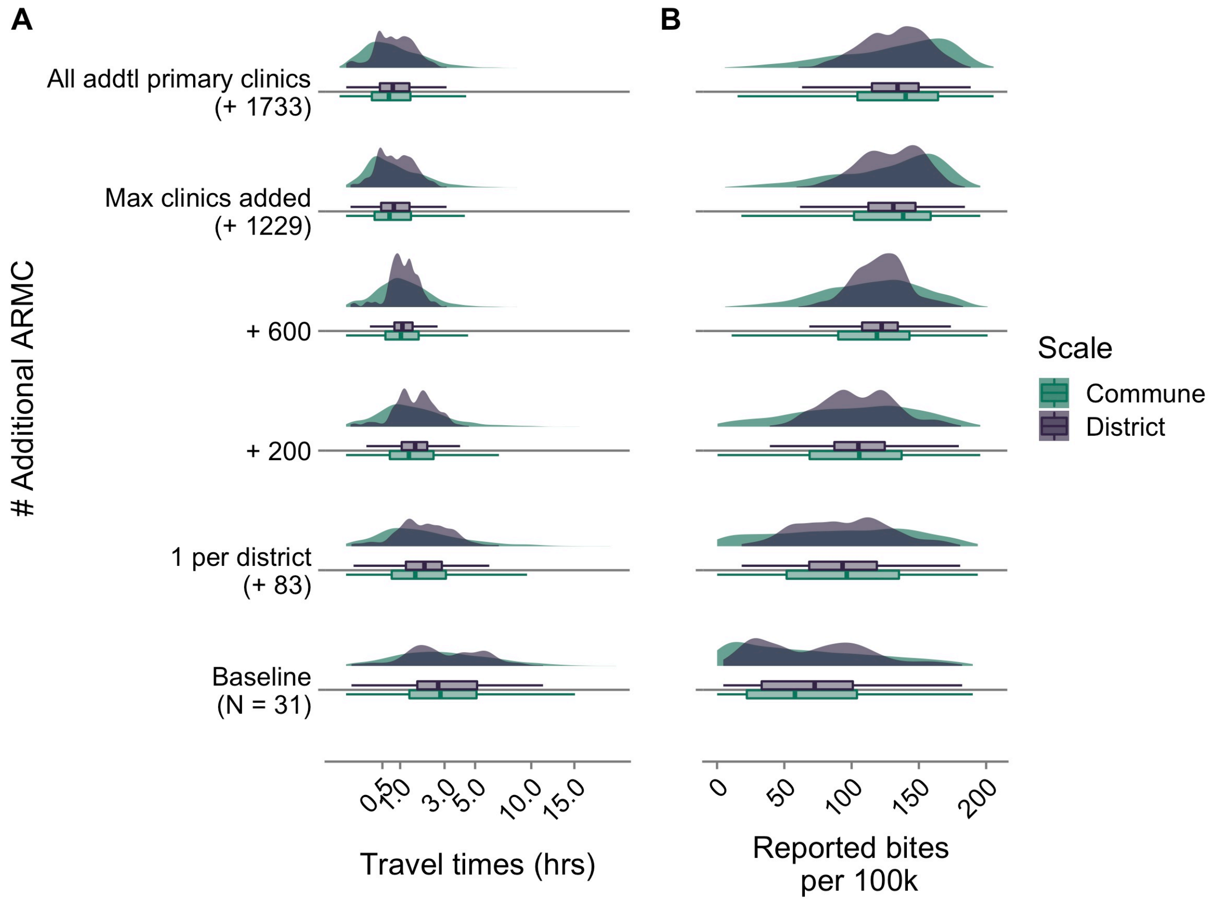

Fig D. Shifts in key metrics as clinics are added.

(A) travel times (hrs, x-axis is square root transformed), (B) bite incidence per 100,000 persons and boxplots showing the median for communes and district models (colors).

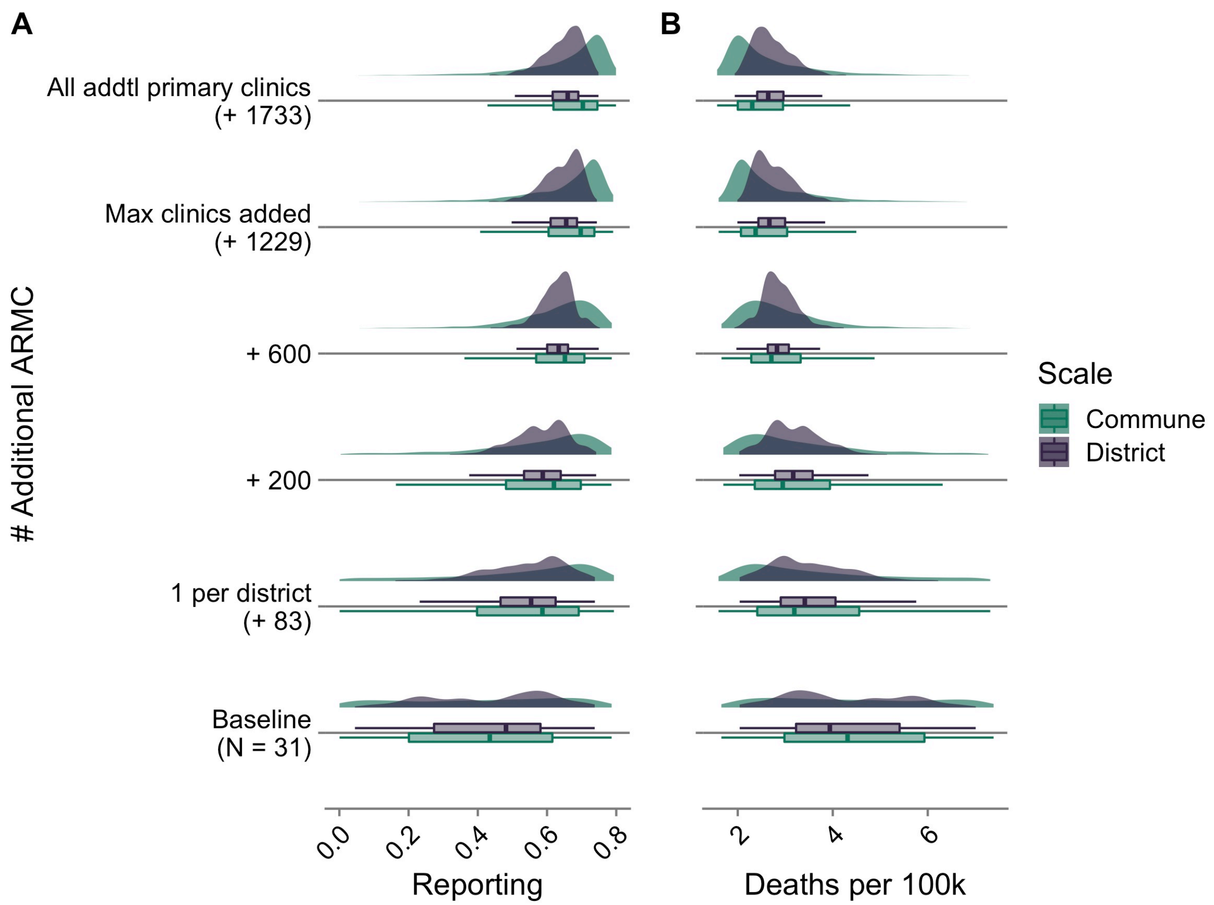

Fig E. Shifts in key metrics as clinics are added.

(A) reporting, (B) death incidence per 100,000 persons for the commune and district models (colors) as clinics are added.

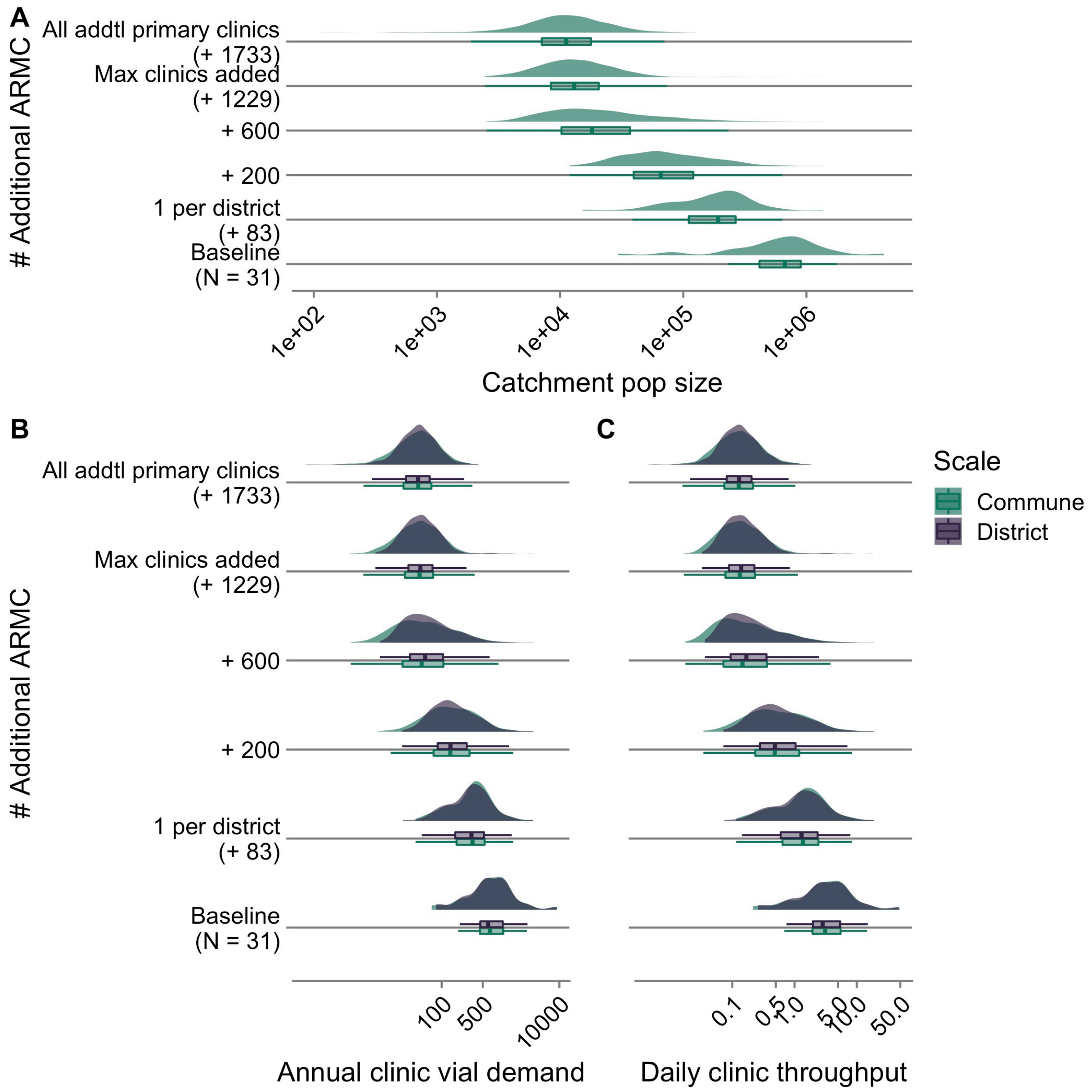

Fig F. Shifts in key metrics as clinics are added.

(A) catchment population size, (B) annual vial demand, and (C) daily throughput (i.e. average number of patients reporting each day) given estimates of bite incidence for the commune and district models (colors). For vial demand estimation, catchment population sizes are the same for each model as these populations are allocated at the grid cell level (i.e. population in a grid cell is allocated to the clinic catchment it is closest to in terms of travel times regardless of district or commune). All x-axes are log transformed.

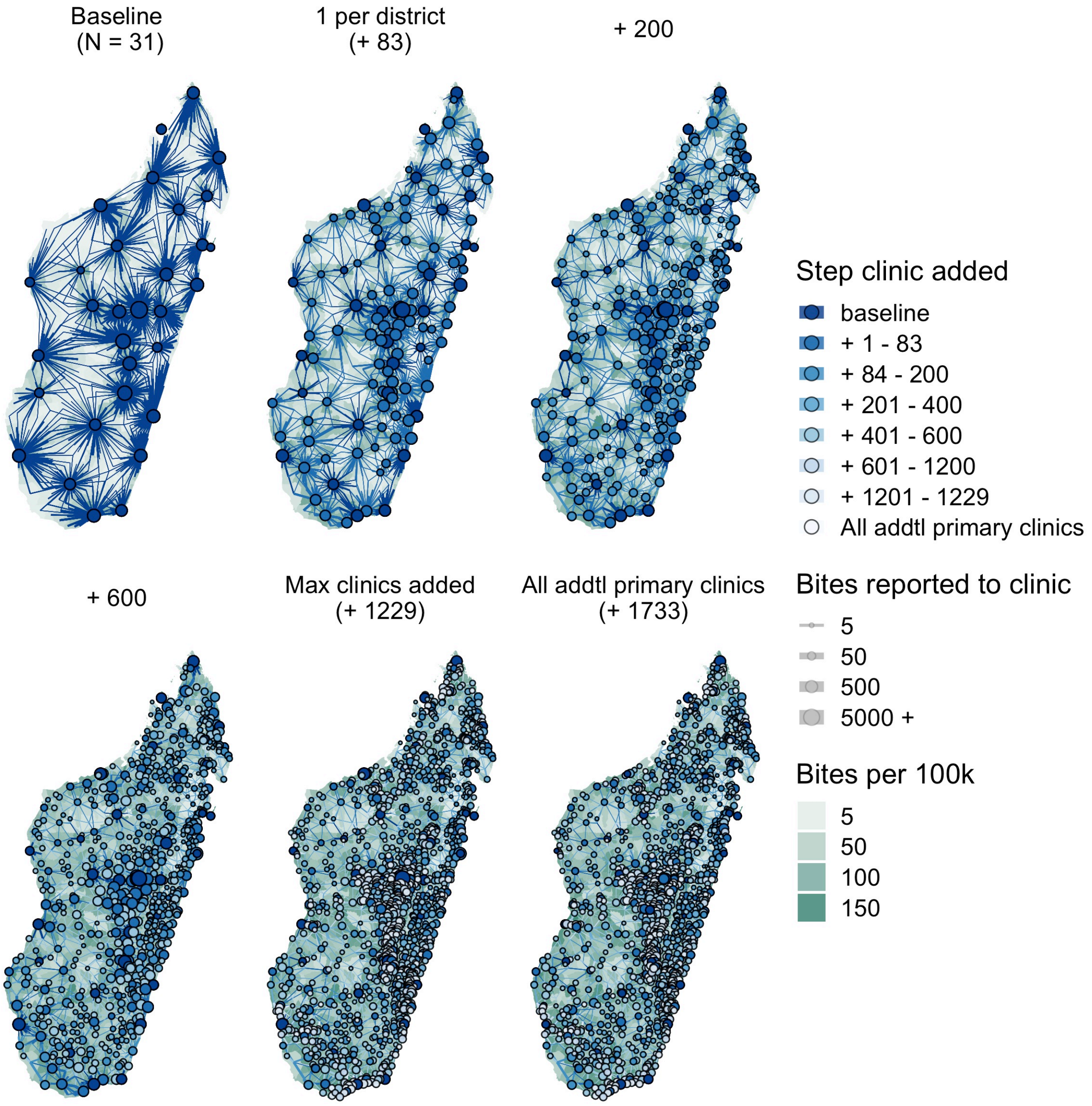

Fig G. Shifts in where bites are reported to as clinics are added for the commune model.

The circles show the clinic locations for each scenario, with size proportional to the annual average bites reported to that clinic. Lines show where the bites are reported from (commune centroid) also proportional to the number of bites. The polygon shading shows the commune level reported bite incidence. Administrative boundaries from OCHA via HDX (https://data.humdata.org/dataset/madagascar-administrative-level-0-4-boundaries, CC-BY-IGO).

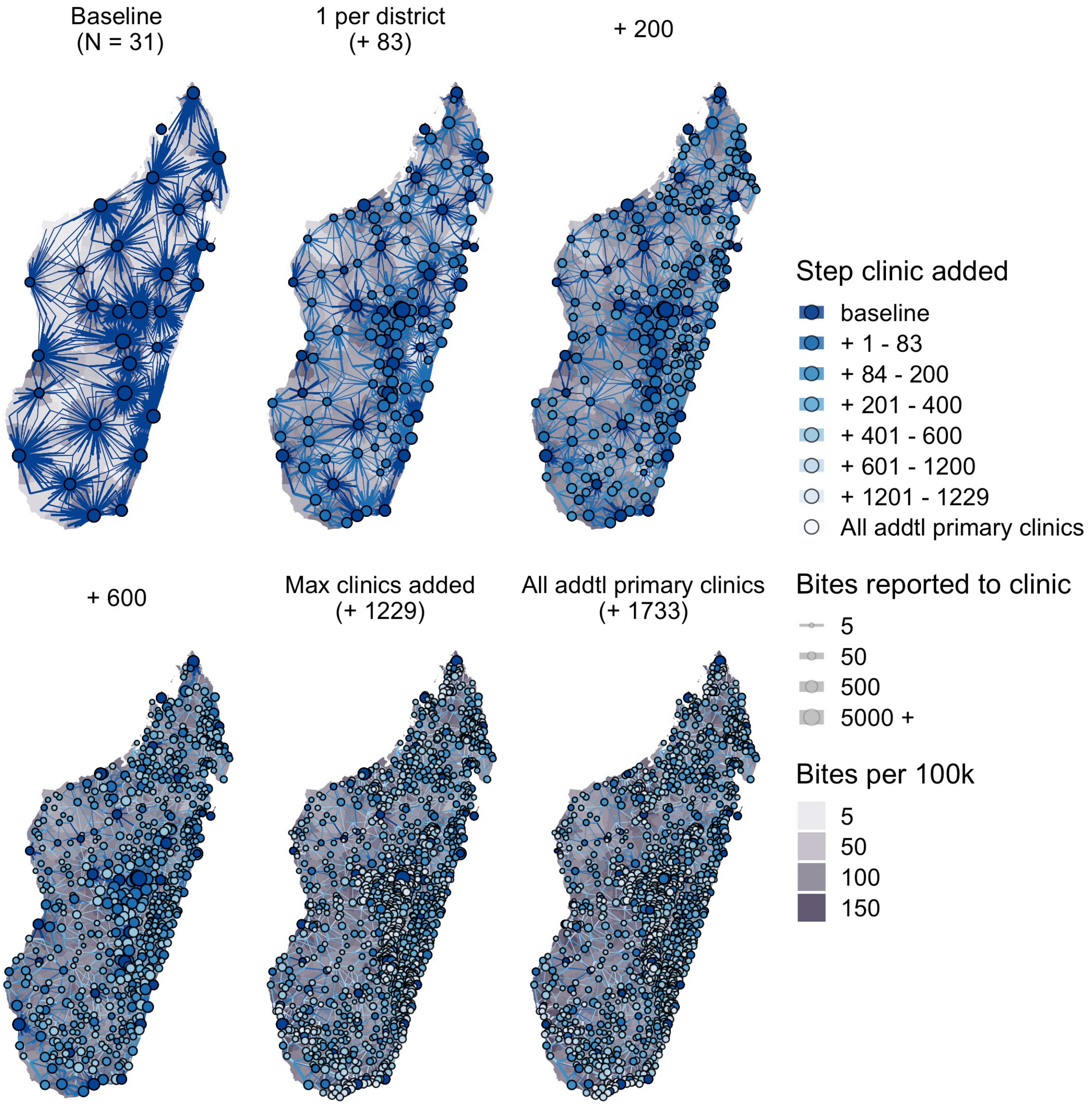

Fig H. Shifts in where bites are reported to as clinics are added for the district model.

The circles show the clinic locations for each scenario, with size proportional to the annual average bites reported to that clinic. Lines show where the bites are reported from (commune centroid) also proportional to the number of bites. The polygon shading shows the district level reported bite incidence. Administrative boundaries from OCHA via HDX (https://data.humdata.org/dataset/madagascar-administrative-level-0-4-boundaries, CC-BY-IGO)
