# Supplementary material for: How geographic access to care shapes disease burden: The current impact of post-exposure prophylaxis and potential for expanded access to prevent human rabies deaths in Madagascar
Source: PLoS Negl Trop Dis. 2021 Apr 26;15(4):e0008821. doi: 10.1371/journal.pntd.0008821 (PMC8102000; doi:10.1371/journal.pntd.0008821)
Supplement: S6 Text — (A) Range of parameters used in the univariate sensitivity analysis (upper 97.5% and lower 2.5% credible interval of posterior for the parameters in the bite incidence model, 95% CI of estimate from [1] for ρmax, and fixed at the minimum and maximum of the range used in the main analysis for all other parameters) (B) Range of estimates of the estimate average deaths at the national level for range of parameter estimates (point shows the estimate presented in the main analyses and the ends show the upper and lower estimates for the parameter range) and (C) estimates for the predicted relationship between travel times and incidence of human rabies deaths across the same parameter ranges (line shows the estimate presented in the main analyses and the envelope shows the upper and lower estimates for the parameter range) for the two models (colors). Fig B. Sensitivity analysis for PEP expansion. (A) Estimates of the maximum proportion reduction of deaths from the baseline for range of parameter estimates (point shows the estimate presented in the main analyses and the ends show the upper and lower estimates for the parameter range) and (B) estimates for how human rabies decreases proportional to the baseline as clinics are added for these same parameter ranges (line shows the mean estimate presented in the main analyses and the envelope shows the mean estimates for the parameters fixed at the upper and lower range) for the two models (colors). See Fig A for ranges used for each parameter. Fig C. Sensitivity analysis for vial demand. (A) Estimates of the baseline vial demand for where each parameter was set to the upper and lower estimate of parameter estimates (point shows the estimate presented in the main analyses and the ends show the upper and lower estimates for the parameter range) and (B) estimates for how vial demand increases as clinics are added for these same parameter ranges (line shows the mean estimate presented in the main analyses and the envelope shows t [file pntd.0008821.s006.pdf]

S6 Text. Sensitivity of burden estimates to parameter assumptions

How geographic access to care shapes disease burden: the current impact of post-exposure prophylaxis and potential for expanded access to prevent human rabies deaths in Madagascar

Rajeev et al. 2021

Table of Contents

Fig A. Sensitivity analyses for baseline burden estimates. 2
Fig B. Sensitivity analysis for PEP expansion. 4
Fig C. Sensitivity analysis for vial demand. 5
Fig D. Sensitivity analysis for exposure scaling with population. 6
References 7

Correspondence: Rajeev et al. 2021 <mrajeev@princeton.edu>

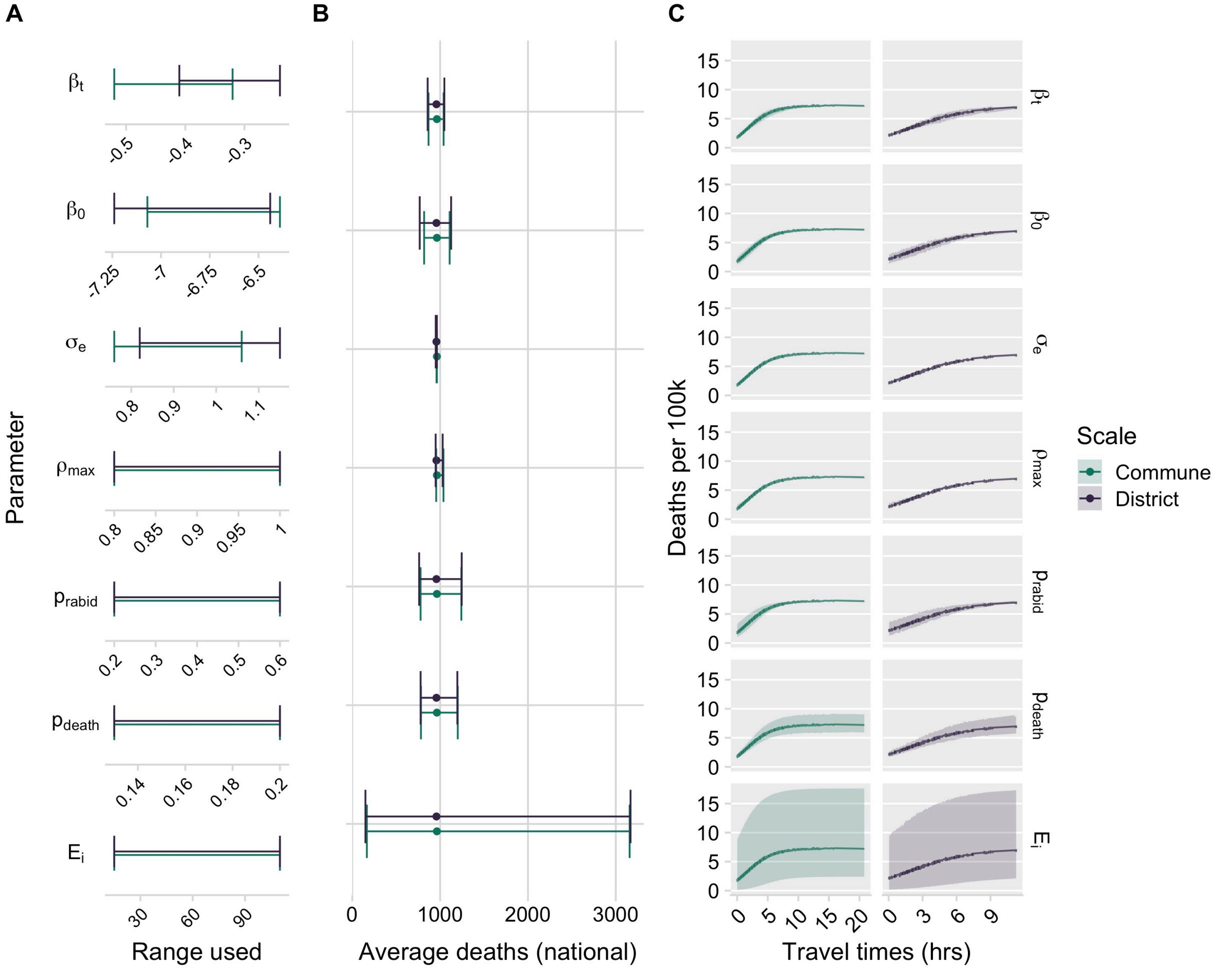

Fig A. Sensitivity analyses for baseline burden estimates.

(A) Range of parameters used in the univariate sensitivity analysis (upper 97.5% and lower 2.5% credible interval of posterior for the parameters in the bite incidence model, 95% CI of estimate from [1] for ρ_{max}, and fixed at the minimum and maximum of the range used in the main analysis for all other parameters) (B) Range of estimates of the estimate average deaths at the national level for range of parameter estimates (point shows the estimate presented in the main analyses and the ends show the upper and lower estimates for the parameter range) and (C) estimates for the predicted relationship between travel times and incidence of human rabies deaths across the same parameter ranges (line shows the estimate presented in the main analyses and the envelope shows the upper and lower estimates for the parameter range) for the two models (colors).

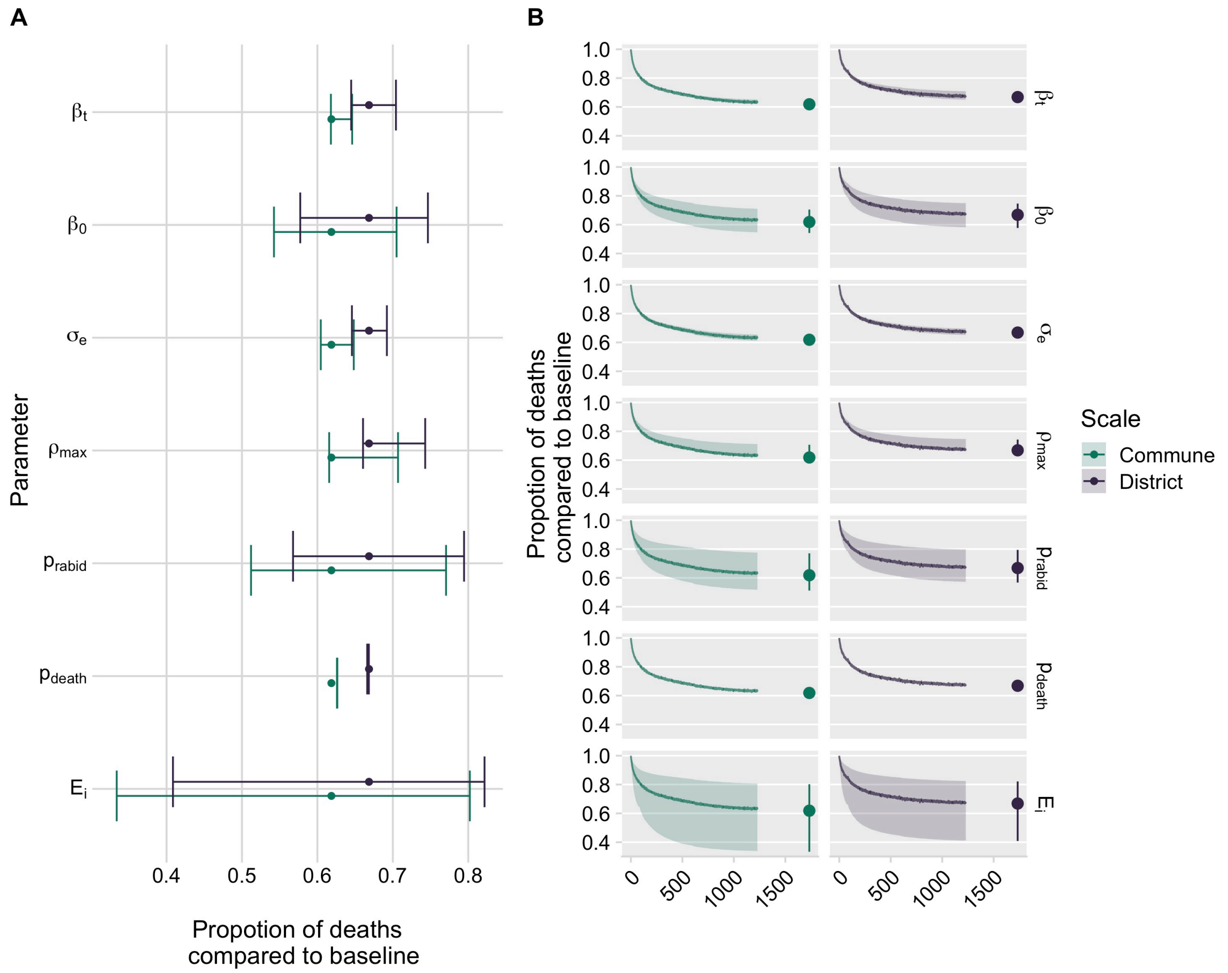

Fig B. Sensitivity analysis for PEP expansion.

(A) Estimates of the maximum proportion reduction of deaths from the baseline for range of parameter estimates (point shows the estimate presented in the main analyses and the ends show the upper and lower estimates for the parameter range) and (B) estimates for how human rabies decreases proportional to the baseline as clinics are added for these same parameter ranges (line shows the mean estimate presented in the main analyses and the envelope shows the mean estimates for the parameters fixed at the upper and lower range) for the two models (colors). See Fig A for ranges used for each parameter.

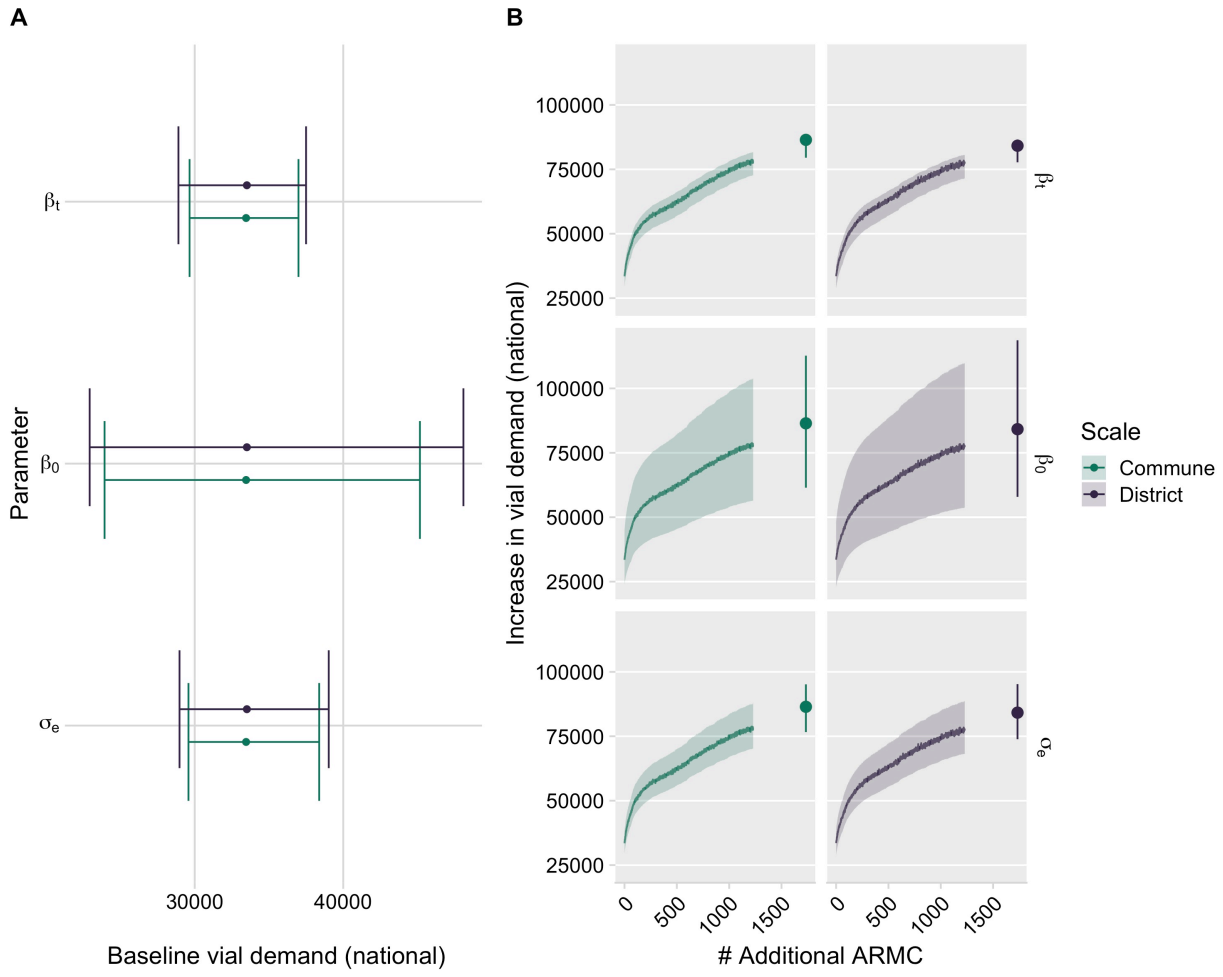

Fig C. Sensitivity analysis for vial demand.

(A) Estimates of the baseline vial demand for where each parameter was set to the upper and lower estimat of parameter estimates (point shows the estimate presented in the main analyses and the ends show the upper and lower estimates for the parameter range) and (B) estimates for how vial demand increases as clinics are added for these same parameter ranges (line shows the mean estimate presented in the main analyses and the envelope shows the mean estimates for the parameters fixed at the upper and lower range) for the two models (colors) for the two models (colors). See Fig A for ranges used for each parameter.

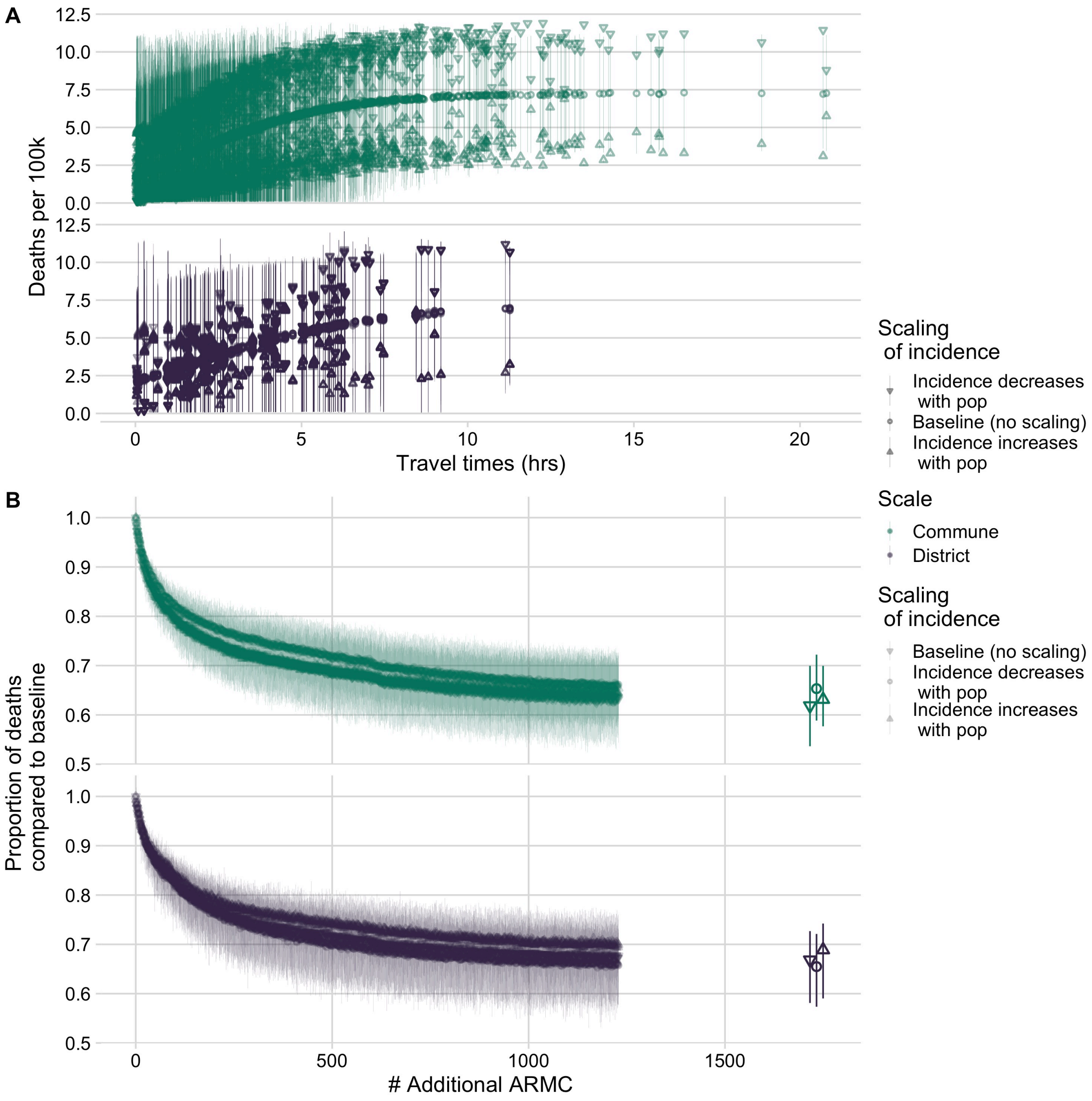

Fig D. Sensitivity analysis for exposure scaling with population.

Predicted relationship between (A) travel times and rabies death incidence per 100k persons and (B) the proportional reduction in deaths from the baseline as clinics are added for the district model vs. commune models (colors) with columns comparing the baseline (presented in main analyses) and assumptions of rabies exposure incidence increasing or decreasing with human population size (shapes). The points show the mean estimates from 1000 simulations and the line ranges show the 95% prediction intervals.

References

1. Changalucha J, Steenson R, Grieve E, Cleaveland S, Lembo T, Lushasi K, et al. The need to improve access to rabies post-exposure vaccines: Lessons from Tanzania. Vaccine. 2019;37: A45–A53.
