# Supplementary material for: How geographic access to care shapes disease burden: The current impact of post-exposure prophylaxis and potential for expanded access to prevent human rabies deaths in Madagascar
Source: PLoS Negl Trop Dis. 2021 Apr 26;15(4):e0008821. doi: 10.1371/journal.pntd.0008821 (PMC8102000; doi:10.1371/journal.pntd.0008821)
Supplement: S7 Text — (PDF) [file pntd.0008821.s007.pdf]

S7 Text. Details on software packages used

How geographic access to care shapes disease burden: the current impact of post-exposure prophylaxis and potential for expanded access to prevent human rabies deaths in Madagascar

Rajeev et al. 2021

Table of Contents

References.. 1

Correspondence: Rajeev et al. 2021 <mrajeev@princeton.edu>

Our analyses relied on a number of open-source software packages for the `R` programming language [1] including the following: `coda` [2], `cowplot` [3], `data.table` [4], `doParallel` [5], `doRNG` [6], `dplyr` [7], `fasterize` [8], `forcats` [9], `foreach` [10], `gdistance` [11], `ggbeeswarm` [12], `ggforce` [13], `ggh4x` [14], `ggplot2` [15], `ggridges` [16], `ggsn` [17], `glue` [18], `here` [19], `igraph` [20], `iterators` [21], `lubridate` [22], `magrittr` [23], `Matrix` [24], `patchwork` [25], `raster` [26], `readr` [27], `rgdal` [28], `rjags` [29], `rmapshaper` [30], `Rmpi` [31], `rngtools` [32], `scales` [33], `sf` [34], `sp` [35], `tidyr` [36], `triangle` [37], `RLang2019` [1]. For full details on implementation please see the archived repositories on Zenodo or on Github.

References

1. R Core Team. R: A language and environment for statistical computing. Vienna, Austria: R Foundation for Statistical Computing; 2019. Available: https://www.R-project.org/

2. Plummer M, Best N, Cowles K, Vines K, Sarkar D, Bates D, et al. Coda: Output analysis and diagnostics for MCMC. 2019. Available: https://CRAN.R-project.org/package=coda

3. Wilke CO. Cowplot: Streamlined plot theme and plot annotations for ’ggplot2’. 2020. Available: https://CRAN.R-project.org/package=cowplot

4. Dowle M, Srinivasan A. Data.table: Extension of ‘data.frame‘. 2020. Available: https://CRAN.R-project.org/package=data.table

5. Corporation M, Weston S. doParallel: Foreach parallel adaptor for the ’parallel’ package. 2019. Available: https://CRAN.R-project.org/package=doParallel

6. Gaujoux R. doRNG: Generic reproducible parallel backend for ’foreach’ loops. 2020. Available: https://CRAN.R-project.org/package=doRNG

7. Wickham H, François R, Henry L, Müller K. Dplyr: A grammar of data manipulation. 2020. Available: https://CRAN.R-project.org/package=dplyr

8. Ross N. Fasterize: Fast polygon to raster conversion. 2020. Available: https://CRAN.R-project.org/package=fasterize

9. Wickham H. Forcats: Tools for working with categorical variables (factors). 2020. Available: https://CRAN.R-project.org/package=forcats

10. Revolution Analytics, Weston S. Foreach: Provides foreach looping construct.

11. van Etten J. Gdistance: Distances and routes on geographical grids. 2020. Available: https://CRAN.R-project.org/package=gdistance

12. Clarke E, Sherrill-Mix S. Ggbeeswarm: Categorical scatter (violin point) plots. 2017. Available: https://CRAN.R-project.org/package=ggbeeswarm

13. Pedersen TL. Ggforce: Accelerating ’ggplot2’. 2020. Available: https://CRAN.R-project.org/package=ggforce

14. van den Brand T. ggh4x: Hacks for ’ggplot2’. 2020. Available: https://github.com/teunbrand/ggh4x

15. Wickham H, Chang W, Henry L, Pedersen TL, Takahashi K, Wilke C, et al. ggplot2: Create elegant data visualisations using the grammar of graphics. 2020. Available: https://CRAN.R-project.org/package=ggplot2

16. Wilke CO. Ggridges: Ridgeline plots in ’ggplot2’. 2020. Available: https://CRAN.R-project.org/package=ggridges

17. Santos Baquero O. Ggsn: North symbols and scale bars for maps created with ’ggplot2’ or ’ggmap’. 2019. Available: https://CRAN.R-project.org/package=ggsn

18. Hester J. Glue: Interpreted string literals. 2020. Available: https://CRAN.R-project.org/package=glue

19. Müller K. Here: A simpler way to find your files. 2017. Available: https://CRAN.R-project.org/package=here

20. file. SA. Igraph: Network analysis and visualization. 2020. Available: https://CRAN.R-project.org/package=igraph

21. Analytics R, Weston S. Iterators: Provides iterator construct. 2019. Available: https://CRAN.R-project.org/package=iterators

22. Spinu V, Grolemund G, Wickham H. Lubridate: Make dealing with dates a little easier. 2020. Available: https://CRAN.R-project.org/package=lubridate

23. Bache SM, Wickham H. Magrittr: A forward-pipe operator for r. 2014. Available: https://CRAN.R-project.org/package=magrittr

24. Bates D, Maechler M. Matrix: Sparse and dense matrix classes and methods. 2019. Available: https://CRAN.R-project.org/package=Matrix

25. Pedersen TL. Patchwork: The composer of plots. 2020. Available: https://CRAN.R-project.org/package=patchwork

26. Hijmans RJ. Raster: Geographic data analysis and modeling. 2020. Available: https://CRAN.R-project.org/package=raster

27. Wickham H, Hester J, Francois R. Readr: Read rectangular text data. 2018. Available: https://CRAN.R-project.org/package=readr

28. Bivand R, Keitt T, Rowlingson B. Rgdal: Bindings for the ’geospatial’ data abstraction library. 2020. Available: https://CRAN.R-project.org/package=rgdal

29. Plummer M. Rjags: Bayesian graphical models using MCMC. 2019. Available: https://CRAN.R-project.org/package=rjags

30. Teucher A, Russell K. Rmapshaper: Client for ’mapshaper’ for ’geospatial’ operations. 2020. Available: https://CRAN.R-project.org/package=rmapshaper

31. Yu H. Rmpi: Interface (wrapper) to MPI (message-passing interface). 2018. Available: https://CRAN.R-project.org/package=Rmpi

32. Gaujoux R. Rngtools: Utility functions for working with random number generators. 2020. Available: https://CRAN.R-project.org/package=rngtools

33. Wickham H, Seidel D. Scales: Scale functions for visualization. 2020. Available: https://CRAN.R-project.org/package=scales

34. Pebesma E. Sf: Simple features for r. 2020. Available: https://CRAN.R-project.org/package=sf

35. Bivand RS, Pebesma E, Gomez-Rubio V. Applied spatial data analysis with R, second edition. Springer, NY; 2013. Available: https://asdar-book.org/

36. Wickham H. Tidyr: Tidy messy data. 2020. Available: https://CRAN.R-project.org/package=tidyr

37. Carnell R. Triangle: Provides the standard distribution functions for the triangle distribution. 2019. Available: https://CRAN.R-project.org/package=triangle
