# Supplementary material for: How geographic access to care shapes disease burden: The current impact of post-exposure prophylaxis and potential for expanded access to prevent human rabies deaths in Madagascar
Source: PLoS Negl Trop Dis. 2021 Apr 26;15(4):e0008821. doi: 10.1371/journal.pntd.0008821 (PMC8102000; doi:10.1371/journal.pntd.0008821)
Supplement: Attachment — Submitted filename: response_reviewers.pdf [file pntd.0008821.s008.pdf]

Response to reviewers

Malavika Rajeev, Princeton University

1/19/2020

We thank the editor and the reviewers for their constructive feedback and close reading of the manuscript and have revised our manuscript accordingly. Both a document with track changes (of both the manuscript & the supplement) and clean separate documents have been submitted, and we have also verified our figures meet the requirements per the PACE tool. The reviewer comments are below with our response in italics (line numbers refer to combined and tracked document).

Reviewer 1

- The authors performed the well-designed epidemiological analysis; given the distance from the residence to the PEP accessibility and assess the rabies risk. There is only once concern arising how much the prevalence (or exposure to residence) of rabid dogs in each area was included into this study. The authors also pointed out the uncertainty of underlying rabies expose according to the sensitivity analysis. This should be more clearly describe in the manuscript, with a validity of the corresponding parameters used in the current study.

  We thank the reviewer for their helpful feedback. We have revised the methods and results section based on this and another reviewer's feedback. The methods section describing the decision tree now forefronts the parameter estimates with their sources. The results section now maps onto the major findings of each of our analyses, and hopefully clarifies the results of each section. We have also added a sentence to the discussion to regarding the validity of our assumptions (see lines 728 - 730).

- Regarding Lines 275-281, the authors indicate the bases with adequate references how to reach the idea that human-to-dog rabies ratio are both positively and negatively correlated with human populations.

  Thank you.

- Overall, the statements in Conclusion part is adequate and convince readers.

  Thank you.

- In Lines 523-525, the authors discuss the potential risk factor of human rabies in Madagascar. These are very general and commonly argued in anywhere. Don't the authors can find the supportive reasons or data for these assumptions? Specifically, box1 may provide the example of association between area and awareness to the risk. We would like to know how these gaps may arise.

We have reorganized this discussion point, and point to a recent KAP study in the Moramanga District that identified such barriers to PEP (see lines 769 - 771). We also discuss the challenges in identifying these risk factors and link this to the case studies in Box 1 (lines 777 - 783).

Reviewer 2

- The authors describe an approach to model the efficacy of different PEP distribution scenarios scenarios interesting and new. The approach is based on several steps and each steps is complex and relies on a set of assumptions. Because it would be difficult to collect empirical data (e.g. in a cluster randomized trial), I think the study is worth publishing. I appreciate that a lot of details and supplementary information is provided and that the source code and data is public. My main critic points are: i) due to the complexity and amount and diversity of information (the appendix alone has 47 pages) the authors should guide the reader better through the manuscript. Some suggestions are provided below, ii) the results are based on many assumptions – I am not sure if the authors are not a bit too optimistic with their approach. I think the next step should be to try to validate the model before "Our framework could be used to guide PEP expansion and improve targeting of interventions."

  We thank the reviewer for their helpful feedback and have revised the manuscript accordingly (see specific points regarding revisions to the methods and results in response to points below). Additionally, we have revised the text in the abstract (lines 48 - 51) and in the conclusion (line 896) to state that in the context of countries becoming eligible for GAVI expansion, this framework may be a useful way to move forward in the absence of baseline data on human rabies risk, and surveillance should be improved to evaluate the impacts of expanding PEP. We also added a point that in other settings, addressing other barriers to PEP access may be more effective (i.e. direct costs of PEP in settings that charge for PEP) (see lines 832 - 835 in the 'Broader context' section of the discussion).

- Ideas to guide the reader a bit better through the manuscript: - One weakness of the paper is that the reader has to jump back and forth to get all the information. Different parameters are introduced over 5 pages. Maybe the method section could be rearranged and a bit sharpened. E.g. p[rabid] is introduced in line 201. In line 208 (a figure legend) it is explained that R[rabid] is constrained. In line 225 it is explained which range was used to express uncertainty about P[rabies], in line 229 the constraining formula is provided and finally in Table 1 the values distribution and reference for this parameter is provided. I don't know if the constraint is so important that it has to be mentioned to this extend. ;Maybe a footnote in table1 would be sufficient. If not, there is certainly a better way to arrange the method section that it can be introduced at only one (maybe 2) occasion.

  We have revised the methods section accordingly (lines 250 - 293). Now all parameters are introduced in Table 1 with their sources and details, and Figure 1 describes how they are used in the decision tree framework. We have moved Table 1 up to the top of the section and describe the constraint on p[rabid] in a footnote and removed this

from the figure description. The minor details about both p[rabid] and rho[max] are now described in the `Description` *column of the table.*

- Same with bite incidence. The result section shows some graphs and a lot of information related to model fit. But the reader is likely more interested in the estimated mean incidence. But this value is reported is not in this but in the next paragraph.

  For the results section, we have revised each section to better state the main finding up front and focus on details in subsequent paragraphs. We combined the sections describing the relationship between bite incidence and travel times and moved some of the details regarding bite incidence estimation to the Supplementary Appendix section S2 and some of the details on model fit and comparisons to section S3.

- Terminology could be simplified. E.g. using consistently either commune or CSB2 in results and discussion to describe this level.

  We have removed acronyms as much as possible, now referring to CSB-II as primary clinics, CSB-I as secondary clinics, and use the term clinics provisioning PEP or alternatively PEP clinic throughout to refer to health facilities provisioning PEP. We've also tried to clarify when using commune and district that we are referring to administrative units.

- The appendix is quite long. A table of content would be helpful

  We added a ToC (not included in the tracked version, but included in the separate clean supplement resubmitted).

- Isn't it possible to integrate the information of tables S6.1/2 into figures S6.1/2.

  We added a panel into Figure S6.1 combining the information from Tables S6.1/2 and removed the tables.

- The most important result is "Estimating the impact of PEP provisioning." I think it would be good to have these results summarized in a table. This table could include/replace table 2.

  We include an estimate of the deaths averted under current PEP provisioning in Table 2 and clarified the column names to make this more clear.

- Out-of-stock is briefly mentioned in this section but I think it is worth to discuss also that the supply chain is getting much more complicated if PEP hast o be delivered to much more clinics.

  We expand on this later in the discussion, and we have rephrased the sentence in the discussion to point directly to complications in the supply chain. We also point to potential solutions for managing these supply chains (i.e. piggybacking on EPI programs, see lines 855 - 857).

- Abstract - After reading the abstract I had no clear impression what the authors exatly did, e.g. ehat kind of data they used to estimate travel time, bite incidence and rabies deaths.

  We have revised the abstract to more clearly describe the data and methods we used (lines 48 - 50).

- "expanding PEP to one clinic per district could reduce deaths by 19%" Please add the number of districts (114) or the number of additional clinics (83).

  Done.

- Authors summary " but our results suggest that expansion alone will not eliminate deaths." Well, how could PEP eliminate human rabies deaths? It does not interrupt transmission. I think this statement should be rephrased.

  We have rephrased this to point to the fact that PEP expansion alone will be unlikely to result in the goal of zero human rabies deaths by 2030, and are trying to communicate to policy makers that countries will need to invest in dog vaccination if this goal is to be achieved (lines 70 - 75).

- Travel time: The authors used geographical information to estimate theoretical travel time as an proxi for access. In addition they tried to validate the approach by comparing the estimates with travel time reported by patients and by travel time reported by the Institute Pasteur. However, very basic information is lacking, e.g. I could even not find the number of patients interviewed and it remains unclear what " driving times collected by IPM during field missions " actually means. Of course, reported and estimated travel times are correlated – both are associated with distance – but if I look at figure 2c or the appendix the variation is remarkable. Some estimate of fit would be nice. Maybe mean/median residual or something similar. By the way: I don't think the shaded areas (CIs around the regression lines) does not add a lot tot he interpretation. I would remove them because the points below are difficult to see.

  We have clarified both the methods and added sample sizes(lines 171 - 187), as well as the results the results (lines 404 - 432) based on the reviewer's helpful feedback. We point to some reasons for the observed variance in the results (lines 421 - 436) and in the discussion (lines 706 - 717), and a co-author and others are working on developing improved friction surfaces that could help better capture travel times based on Madagascar specific data. We also touch briefly on the issues with the data we used to 'ground-truth' travel times from the friction surface in the discussion (lines 718 - 720). See Table S1.1 for R^2 estimates for the linear models we used to compare the metrics, which we now also report in the main results text (line 381). We removed the CIs around the regression lines for both Fig 2C and Fig S1.4.

- Figure2.2: A indicates " (A) The daily time series of the number of forms submitted by each clinic," I don't think that the figure presents time series data. B boxplots for

4 points are (literally?) a bit pointless. Present only the 4 points or the 4 points + mean or median.

*Thanks for **pointing** this out, fig S2.2 and it's caption have been revised accordingly, with S2.2A showing the number of forms submitted for each clinic with excluded periods shown in grey and S2.2B showing the four point estimates of reporting and the line showing the range.*

Minor points :

- Is the overdisperion parameter presented in Fig 4 the estimate or log(estimate)? If it is the estimate, an estimate below 1 is rather unusual.

  The overdispersion estimate is not on a log scale, but we incorrectly describe it's form in the methods and have corrected it. The overdispersion parameter is the standard deviation of a random variable with mean 0 (see corrected lines 241 - 243) and it is accordingly scaled by the offset (population size). This means that the estimated overdispersion is actually quite large, as described in the text and vizualised in the figures. For example, given a travel time of zero with a population of 100,000 and taking approximately the mean of the posteriors, we would predict exp(-5.5 +/- 0.9)100000 = 170 - 1000 bites per 100,000 persons. We apologize for the error in the text and thank the reviewer for catching it!

- Lines 131 133: please use superscript instead of caret to indicate exponentiation

  Corrected.

- Lines 493: 45%%

  Corrected.

- Lines 494-494: Please keep the same order.

  Corrected.

Reviewer 3

- The analysis presented match the analysis plan and the results are clearly and completely presented. The figures have sufficient quality.

  Thank you.

- All the criterias are met. Minor points are the following: - Please indicate in S1.1A that the black crosses represent ARMCs.

  Done.

- Delete in line 132 'the'

  Corrected

- Fig. 1 with its description is confusing. I would rather recommend a list with the variables and their description.

  We have revised this section based on this and another reviewers feedback (lines 250 - 293). Briefly, we moved the table with the parameters and description to the top of the section, use the figure to show how these parameters were used, and additional choices regarding parameter ranges in the subsequent paragraph.

- Line 234, can this really be assumed? A baseline study in West Africa demonstrated, that only half of the bite victims complete PEP. However, based on the information regarding the high patient compliance in Madagaskar (line 539), I think you can make this assumption here.

  Thank you for pointing this out, we have moved the reference from line 539 to the methods (now line 291 - 293) to ground this assumption up front.

- Remove a % in line 493

  Corrected.

- This work nicely demonstrates that even with provisioning PEP all over the country, there will still be rabies deaths in the country. So besides the provision of PEP for humans, the disease needs to be eliminated in the animal reservoir.

  Thank you, we've also revised these points based on the other reviewers feedback to address that PEP alone is unlikely to get countries to the goal of 'Zero by 30' (the WHO goal of zero human rabies deaths from dog-mediated rabies by 2030; see lines 96 - 97).

- It was a pleasure to read this fantastic work.

  We thank the reviewer for their kind and helpful review!
